# Lead or cadmium co-contamination alters benzene and toluene degrading bacterial communities

**DOI:** 10.1007/s10532-023-10021-w

**Published:** 2023-02-25

**Authors:** Aniko Konya, Brice A. Fiddler, Olivia Bunch, Kendra Z. Hess, Cade Ferguson, Mark J. Krzmarzick

**Affiliations:** 1 Environmental Science Graduate Program, Oklahoma State University, Stillwater, OK USA; 2 School of Civil and Environmental Engineering, Oklahoma State University, Stillwater, OK USA

**Keywords:** Hydrocarbon degradation, Heavy metal resistance, Microbial diversity, *Ralstonia*, *Cupriavidus*

## Abstract

Co-contamination of hydrocarbons with heavy metals in soils often complicates and hinders bioremediation. A comprehensive characterization of site-specific degraders at contaminated sites can help determine if in situ bioremediation processes are sufficient. This study aimed to identify differences in benzene and toluene degradation rates and the microbial communities enriched under aerobic conditions when different concentrations of Cd and Pb are introduced. Microcosms were used to study the degradation of 0.23 mM benzene or 0.19 mM toluene under various concentrations of Pb (up to 240 µM) and Cd (up to 440 µM). Soil collected from a stormwater retention basin receiving runoff from a large parking lot was utilized to seed the microcosms. The hydrocarbon degradation time and rates were measured. After further rounds of amendment and degradation of benzene and toluene, 16S rRNA gene amplicon sequencing and quantitative PCR were used to ascertain the microbial communities enriched under the various concentrations of the heavy metals. The initial degradation time for toluene and benzene was 7 to 9 days and 10 to 13 days, respectively. Degradation rates were similar for each hydrocarbon despite the concentration and presence of metal co-contaminant, however, the enriched microbial communities under each condition differed. Microcosms without metal co-contaminant contained a diversity of putative benzene and toluene degrading bacteria. Cd strongly reduced the richness of the microbial communities. With higher levels of heavy metals, genera such as *Ralstonia*, *Cupriavidus*, *Azoarcus*, and *Rhodococcus* became more dominant under various conditions. The study finds that highly efficient benzene- and toluene-degrading consortia can develop under variations of heavy metal co-contamination, but the consortia are dependent on the heavy metal type and concentrations.

**Supplementary Information:**

The online version contains supplementary material available at 10.1007/s10532-023-10021-w.

## Introduction

Benzene and toluene are aromatic hydrocarbons and natural components of crude oil and refined petroleum products. When oil and petroleum contamination of soil and groundwater occur, benzene, toluene, and similar compounds are often recalcitrant. Benzene has a low minimum drinking water contamination level and often governs the need for bioremediation. Several studies have identified genera capable of degrading benzene and toluene in polluted environments, such as *Aminobacter*,* Burkholderia*,* Pseudomonas*, *Geobacter*, and *Rhodococcus* (Hendrickx et al. 2006; Lhotský et al. 2017; Sperfeld et al. 2018). A comprehensive characterization of site-specific degraders at contaminated sites helps determine if in situ bioremediation processes are efficient for treatment goals or if the site needs to undergo a biostimulation or bioaugmentation treatment to increase bioremediation rates (Lhotský et al. 2017).

Co-contamination of organic pollutants with heavy metals in soils and groundwater is exceedingly common (Lookman et al. 2004; Madrid et al. 2016; Roane et al. 2001). Petroleum has a natural metal content of up to 10% w/w, which often includes iron, copper, nickel, and vanadium (Speight 2014). Prior to 1980 in the United States (US), lead (Pb) was added to refined petroleum as a standard fuel additive. Additionally, many contaminated sites in the US are subject to independent sources of heavy metal and petroleum contamination, such as industrial facilities, landfills, hazardous waste handling, and disposal sites (Sandrin and Maier 2003; US EPA 2020). The concentration and types of co-contaminants are important to understanding the resulting effects on the hydrocarbon-degrading microbial community (Schwarz et al. 2019).

Heavy metal co-contamination is problematic for bioremediation. High concentrations of Pb and Cd strongly correlate with toxicity, impeding the growth and activity of the hydrocarbon degrading microorganisms. Studies have found that even small amounts of metals can significantly decrease the biodegradation of oil products (Zukauskaite et al. 2008). Research about the impacts of heavy metals on the biodegradation of hydrocarbons such as toluene and benzene is essential for advancing bioremediation strategies (Weelink et al. 2010; Vaezihir et al. 2012).

Our objective was to compare degradation rates and the benzene and toluene degrading consortia under various heavy metal concentrations. The hypothesis was that heavy metal toxicity would result in lower degradation rates, and that unique bacteria consortia would be enriched under different concentrations of Cd and Pb co-contamination. We found that with this soil inoculum, collected from a stormwater drainage basin, the degradation rates and time to completion of benzene and toluene were unimpacted by the metal concentration, however the degradation communities that were enriched in each condition differed. This study shows the facile adaptability of this soil community to heavy metal co-contamination, but with a reduced diversity of putative degraders under heavy metal stressed conditions. This suggests that benzene and toluene degradation in a soil with a diverse population of in situ degraders is more resilient to the negative effects of heavy metal toxicity. Bioremediation in heterogeneous co-contaminated sites necessitates a diversity of hydrocarbon degrading bacteria.

## Materials and methods

### Construction of the aerobic microcosms

One kg of surface soil was collected from a stormwater retention basin receiving parking lot runoff in Stillwater, Oklahoma, USA. Benchtop aerobic microcosms of 160 mL were prepared in silanized serum bottles (see Supplementary Information, Figure S1). Microcosms consisted of 2 g of homogenized soil and 100 mL of an autoclaved mineral medium), modified from Shelton and Tiedje (1984) with trace elements modified from Zehnder and Wuhrmann (1977) (see Supplementary Information). Pb and Cd were amended as cadmium chloride and lead acetate, respectively. The concentration of heavy metals was tested at 0.44, 4.4, 44, and 440 µM of Cd, and 0.24, 2.4, 24, and 240 µM of Pb (corresponding to 50 μg L^−1^ to 50 mg L^−1^ for both heavy metals). Heavy metals-free microcosms were also tested as controls. The microcosms were sealed with Teflon-coated septa and amended with 0.23 mM (18 mg L^−1^) of benzene or 0.19 mM (17.5 mg L^−1^) of toluene with a glass syringe through the septa. Each concentration and type of heavy metal was independently tested with either toluene or benzene as the organic pollutant resulting in 18 conditions. Microcosms were incubated at 30 °C on a shaker table at 50 rpm. Each condition was tested in parallel in triplicate microcosms. Duplicate autoclaved microcosms were also operated under each condition to test for abiotic loss of contaminant. After benzene or toluene degradation was observed, the microcosms were re-opened to refresh headspace O_2_, closed, and re-amended with 0.23 mM benzene or toluene 0.19 mM (respectively). This step was repeated until a total of seven re-amendments were performed.

#### Gas chromatography

Benzene and toluene concentrations were measured using an Agilent 8890 gas chromatograph with a flame ionization detector (GC-FID). For each sampling, a 0.1 mL sample of headspace from the microcosms was extracted through the microcosm septa and injected onto the GC-FID with a gastight syringe. The inlet was operated in splitless mode at 250 °C, the FID detector was set to 300 °C, and the oven temperature was isothermal at 100 °C. External standard curves were made by adding 100 mL of DI water to 160 mL serum bottles, autoclaving them, then adding benzene or toluene via stock solution for the targeted concentration. Five standards were made at aqueous concentrations ranging from 0.011 to 0.45 mM for benzene and 0.009 mM through 0.38 mM for toluene. The syringe was rinsed with hexane and evacuated via heat and vacuum with a Hamilton Syringe Cleaner between injections. Technical duplicates were performed on every measurement. Peak areas for technical duplicates were averaged, the concentration of benzene or toluene was calculated using the standard curve, and first-order degradation rates were calculated by taking the slope of the ln (concentration) versus time data for each individual microcosm. The average and standard deviations of degradation rates and times to complete degradation from the triplicate microcosms for each treatment were calculated and reported below.

#### DNA extraction

DNA extraction was performed on samples saved upon completion of the benzene or toluene re-amendment series. After the final confirmation of benzene or toluene degradation, microcosms were shaken, and 1.5 mL of the mixed slurry was transferred into microcentrifuge tubes via cut-off Pasteur pipettes. The sample was pelleted by centrifugation at 5000 × g for 10 min, and all but 100 µL of supernatant was removed. According to the manufacturer’s protocol, the remaining sample was then subject to DNA extraction with a DNeasy PowerSoil Kit (Qiagen Germantown, MD, USA). Final DNA concentrations were measured in technical triplicate using a Quantus Fluorometer (Promega Corporation, Madison, WI, USA) with dsDNA System reagents following the manufacturer's instruction.

#### 16S rRNA gene microbial community analysis

A total of ten samples were prepared for 16S rRNA gene amplicon sequencing. These samples were from both benzene and toluene degrading microcosms co-contaminated with low (0.24 µM Pb or 0.44 µM Cd) or high (240 µM Pb and 440 µM Cd) heavy metals concentration. Additionally, the control microcosms without heavy metals were sequenced. Aliquots of DNA were mixed in equal concentrations into combined samples for each of these sets of triplicate microcosms. 16S rRNA gene amplification and amplicon sequencing were performed by Molecular Research DNA (Shallowater, TX). Samples were barcoded, and amplification was performed on the V4 variable region of the 16S rRNA gene with universal primers 515F (5′-GTGCCAGCMGCCGCGGTAA-3′) and 806R (5′-GGACTACVSGGGTATCTAAT-3′). Amplicons were sequenced with an Ion Torrent Ion S5 XL next-generation sequencing system. With a proprietary analysis pipeline (MR DNA, Shallowater TX, USA), chimeras, short sequences (< 150 bp), and sequences with ambiguous base calls were removed. Sequences were trimmed of barcodes and primers, and sequences were denoised. OTUs (Operational Taxonomic Unit) were generated by clustering at the 3% divergence level and taxonomically classified with BLASTn against a database derived from RDPII (http://rdp.cme.msu.edu) and NCBI (www.ncbi.nlm.nih.gov). Raw sequences have been uploaded to NCBI’s database under BioProject PRJNA792805. V4 16S rRNA gene sequences are available under accession numbers SAMN24466134-SAMN24466143. The OTU sequences were further analyzed with a phylogenetic analysis performed in MEGA X (Kumar et al. 2018) (See Supplementary Information).

#### Quantitative PCR (qPCR) amplification

Thirty-nine qPCR assays were used to quantify the major OTUs identified from the 16S rRNA gene amplicon analysis. Four assays were previously designed (assays targeting the *Ralstonia* and *Cupriavius* spp. (Lozano et al. 2019), the *Noviherbaspirillum* and *Herbasprillium* spp. (Lozano et al. 2019), *Nitrosospira* spp. (Madeira et al. 2019), and *Hyphomicrobium* spp. (Madeira et al. 2019). The other 35 assays were newly designed for this study. Primers were designed using Primer-BLAST (NIH NCBI) and additionally checked for specificity against the other OTUs in this study using MEGA X (Kumar et al. 2018). Standards were prepared via PCR amplification with DNA template from the microcosms, cloning of cleaned PCR product using the pGEM-T Easy Vector kit (Promega Corporation, Madison, WI), and extraction of plasmids from overnight cultures using the QIAprep Spin Miniprep Kit (Qiagen, Germantown, MD, USA). Plasmids were quantified with Quantus dsDNA kit (Promega Corporation, USA) and serially diluted in 0.5 × TE buffer. Assay information (primers, amplicon size, efficiencies, and linear ranges) is provided in the Supplementary Information.

The qPCR assays were performed with a CFX Connect Real-Time PCR Detection System (Bio-Rad Laboratories, Hercules, CA, USA). Each qPCR reaction totaled 10 μL and contained 1 × iTaq SYBER Green Supermix (Bio-Rad Laboratories, Hercules, CA, USA), 10 μg of bovine serum albumin, 300 nM of each forward and reverse primer, and 1 μL of DNA template. Thermocycling conditions were as follows: 3 min at 95 °C followed by 40 cycles of 30 s at 95 °C and 30 s 59 °C. A melting curve analysis was performed for quality control. Standards and samples were analyzed in triplicate. Samples were quantified with the standard curve, log_10_ transformed, and triplicate readings averaged.

#### Statistical analysis

Student *t*-tests were conducted to test statistical significance between triplicate microcosms using Excel or R version 1.2. Bacterial community compositions were analyzed with non-metric multidimensional scaling (NMDS) using the VEGAN package in R version 1.2. Dendrogram and heatmap analysis was performed with the HEATMAP.2 function with Ward’s minimum variance clustering method (ward.D2) using the GPLOTS package in R version 4.0.4. The dendrogram served to visualize clustering and display the distances among individuals. The data set shown in the heatmap is mean-centered.

## Results

Soil bacteria degraded benzene and toluene in the microcosms at all tested concentrations of Pb and Cd (Table 1). The first order degradation rates of benzene ranged from 0.28 to 0.39 day^−1^ and complete degradation occurred between 10 and 13 days. For toluene, the degradation rates were generally higher than for benzene, ranging from 0.37 to 0.51 day^−1^. Complete degradation of toluene was observed between 7 and 9 days. No lag time was observed for benzene degradation. Many, but not all, microcosms had a 1-day lag time degrading toluene. Autoclaved controls did not show either benzene or toluene loss from volatilization or abiotic degradation. During the seven rounds of hydrocarbon re-amendment, the complete degradation of benzene and toluene under all metal conditions occurred slightly quicker. By the seventh re-amendment, complete degradation in the benzene microcosms occurred within ten days after reamendment, while the toluene degradation quickened to within 2 days.

**Table 1:** Degradation rates and lag times of benzene and toluene in initial microcosms

Microcosm treatment	Degradation rates (day^−1^)	Time to complete degradation (days)
0.23 mM Benzene (no metals)	0.38 ± 0.05	13 ± 0
0.23 mM Benzene + 0.44 µM CdCl_2_	0.32 ± 0.09	11 ± 4
0.23 mM Benzene + 4.4 µM CdCl_2_	0.35 ± 0.07	12 ± 2
0.23 mM Benzene + 44 µM CdCl_2_	0.38 ± 0.08	12 ± 2
0.23 mM Benzene + 440 µM CdCl_2_	0.35 ± 0.06	13 ± 0
0.23 mM Benzene + 0.24 µM Pb(CH_3_COO)_2_	0.28 ± 0.08	12 ± 2
0.23 mM Benzene + 2.4 µM Pb(CH_3_COO)_2_	0.36 ± 0.10	13 ± 0
0.23 mM Benzene + 24 µM Pb(CH_3_COO)_2_	0.28 ± 0.06	12 ± 2
0.23 mM Benzene + 240 µM Pb(CH_3_COO)_2_	0.39 ± 0.05	10 ± 3
0.19 mM Toluene (no metals)	0.43 ± 0.11	7 ± 0
0.19 mM Toluene + 0.44 µM CdCl_2_	0.40 ± 0.04	8 ± 1
0.19 mM Toluene + 4.4 µM CdCl_2_	0.39 ± 0.05	7 ± 0
0.19 mM Toluene + 44 µM CdCl_2_	0.50 ± 0.12	7 ± 0
0.19 mM Toluene + 440 µM CdCl_2_	0.47 ± 0.03	8 ± 1
0.19 mM Toluene + 0.24 µM Pb(CH_3_COO)_2_	0.37 ± 0.09	9 ± 0
0.19 mM Toluene + 2.4 µM Pb(CH_3_COO)_2_	0.51 ± 0.08	8 ± 1
0.19 mM Toluene + 24 µM Pb(CH_3_COO)_2_	0.45 ± 0.07	8 ± 1
0.19 mM Toluene + 240 µM Pb(CH_3_COO)_2_	0.46 ± 0.07	8 ± 1

**Fig. 1:**
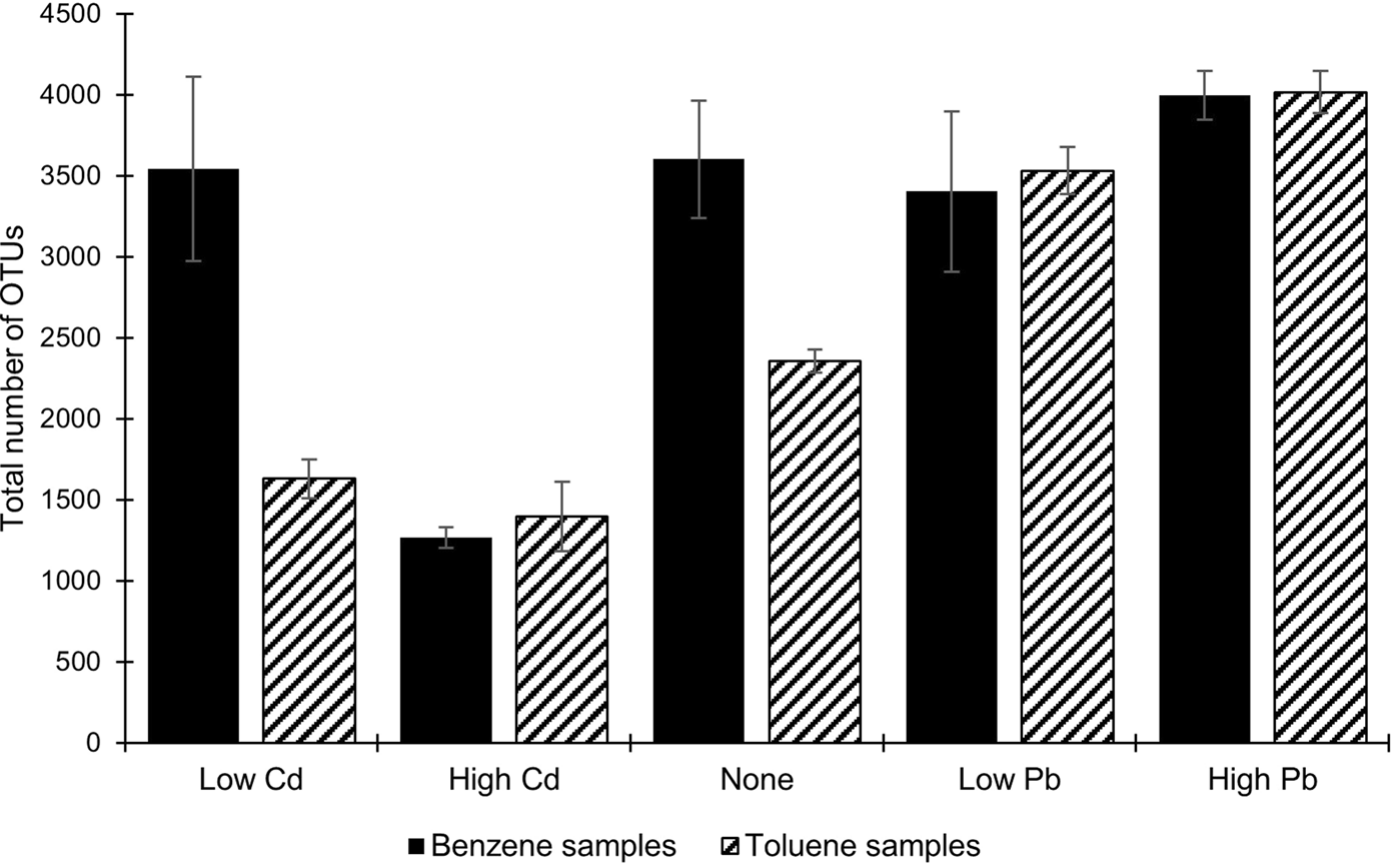
The total number of OTUs found in each sample from 16S rRNA gene amplicon sequencing. Low concentration refers to 0.24 µM Pb or 0.44 µM Cd. High concentration refers to 240 µM Pb or 440 µM Cd. None indicates the samples, which do not have any heavy metals

**Fig. 2:**
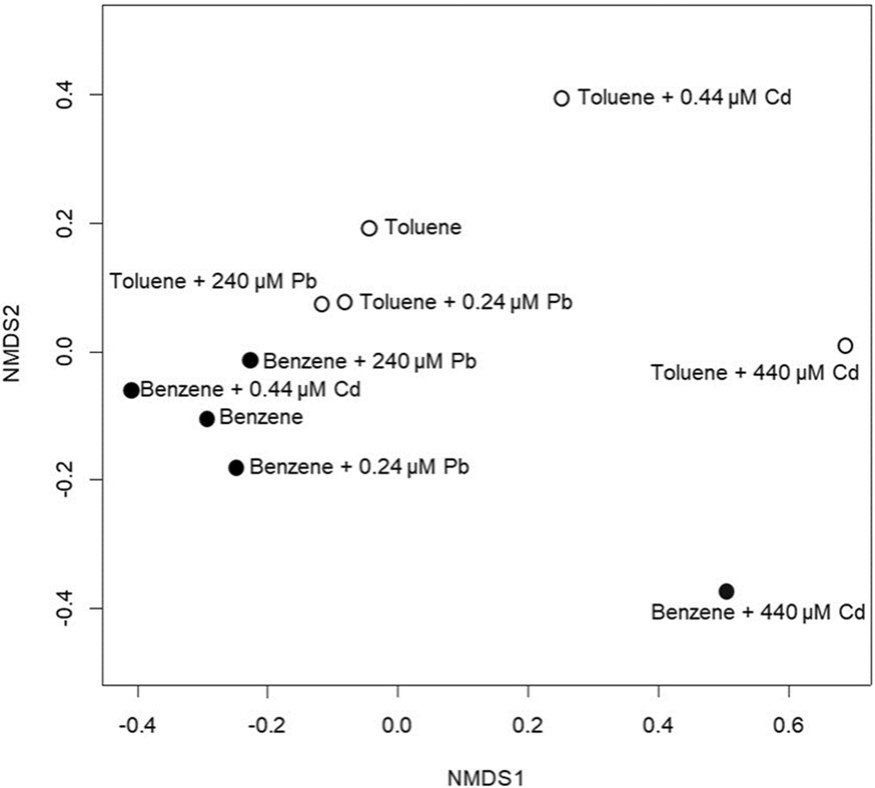
Results of the non-metric multidimensional scaling (NMDS) analysis of bacterial community composition as determined by 16S rRNA gene amplicon sequencing

At the end of the seventh re-amendment of benzene or toluene, the microcosms were subject to microbial community analysis. An initial assessment was performed with 16S rRNA gene amplicon sequencing. Combined DNA extracts from the triplicate sets for both benzene and toluene degrading cultures with low (0.24 µM Pb or 0.44 µM Cd), high (240 µM Pb or 440 µM Cd), and no heavy metals were analyzed and between 43,537 and 209,190 sequences were obtained per sample. A total of 7,165 OTUs (operational taxonomic units) were identified in the dataset. Samples contained between 1,267 and 4,016 OTUs. For both toluene and benzene degrading communities, high Cd co-contaminant resulted in much lower numbers of OTUs (Fig. 1). The cultures with high Pb co-contaminants contained the highest numbers of OTUs, possibly due to the additional carbon source (acetate) amended with the metal. A non-metric multidimensional scaling (NMDS) analysis of bacterial community composition was performed with the 16S rRNA gene amplicon sequencing analysis. The microbial communities co-contaminated with Cd diverged prominently and uniquely from the communities with Pb or no heavy metal (Fig. 2).

**Fig. 3:**
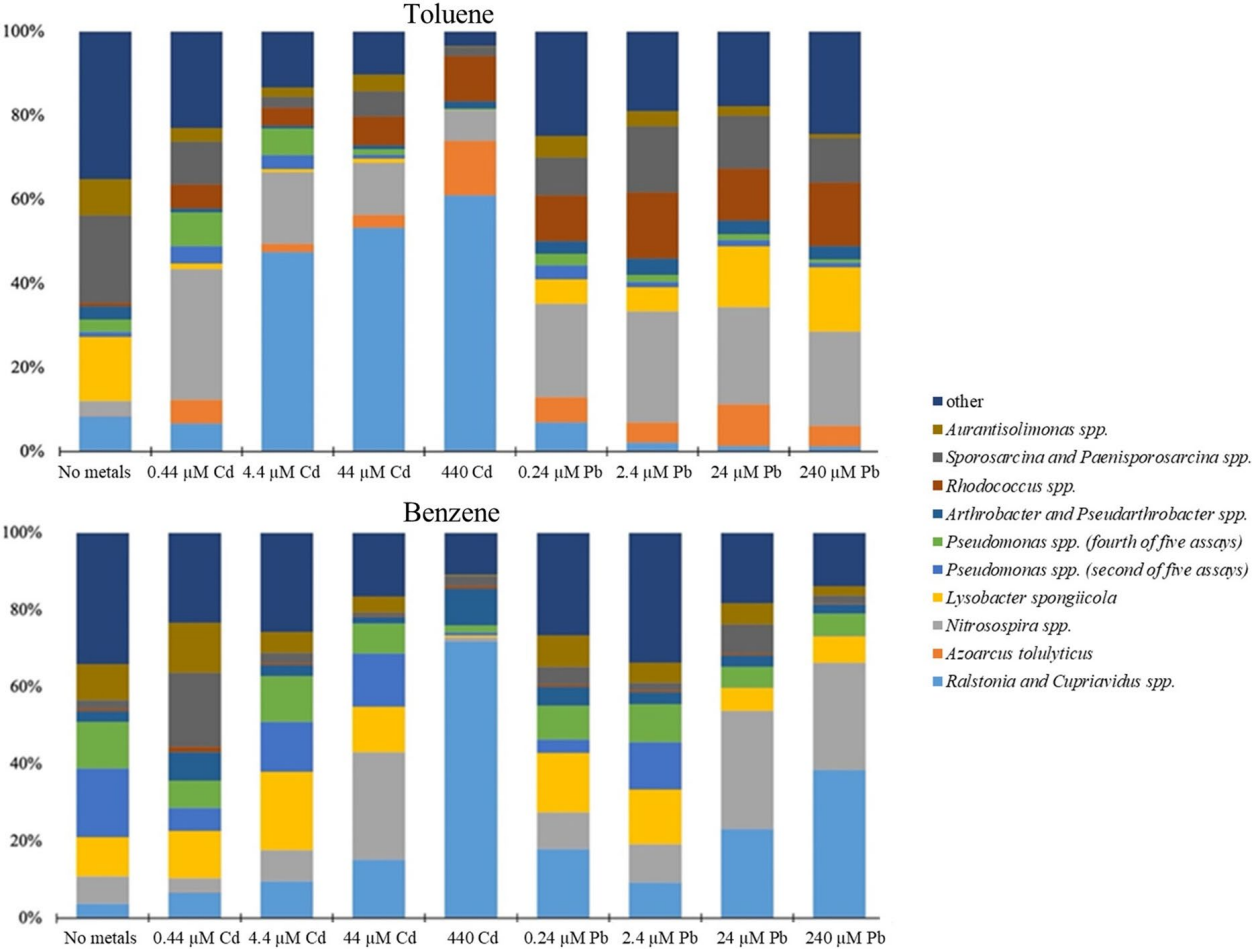
Partial results of the qPCR data analysis to demonstrate the dominant *Bacteria* in the microcosms. Shown is the % of 16S rRNA genes measured for the phylogenetic group as a total of 16S rRNA genes measured in all 39 assays for those microcosms. Full results of qPCR data are shown in the Supplementary Information

These results indicated that though degradation rates were similar, the microbial communities that developed were unique. Thus, a set of qPCR assays targeting the dominant community members identified from the amplicon sequencing were developed. These qPCR assays measured 39 phylogenetic groups amongst replicate microcosms and at all concentrations of heavy metals tested. Full results of these qPCR assays are present in the Supplementary Information (Tables S5 to S8), with summary results shown of most dominant community members shown in Fig. 3 (expressed as % 16S rRNA genes of the total 16S rRNA genes measured). The qPCR assays confirmed the alteration in benzene and toluene degrading bacterial communities when lead or cadmium co-contamination was present.

The co-contamination of 0.44 µM of Cd in the toluene degrading microcosms resulted in a significant decrease of 10 measured phylogenetic groups, and 440 µM of Cd led to a significant decrease of 17 of the groups. The three most abundant groups in the toluene-degrading control microcosms (no metal added) were a group of *Sporosarcina* and *Paenisporosarcina* spp., *Lysobacter spongiicola*, and *Aurantisolimonas* spp. These groups were all significantly lower in the microcosms with Cd co-contamination (Student t-test, P < 0.05). The *Sporosarcina* and *Paenisporosarcina* group was 6.96 ± 0.08 log_10_ units of 16S rRNA genes mL^−1^ in the toluene-degrading microcosms without Cd and was marginally lowered to 6.35 ± 0.22 log units in the microcosms with 0.44 µM Cd. Higher amounts of Cd did not significantly decrease this abundance further. *Lysobacter spongiicola* and *Aurantisolimonas* spp. both showed more marked decreases in abundance and with larger decreases at higher Cd levels. *Lysobacter spongiicola*, for example, was 6.82 ± 0.13 log10 units of 16S rRNA genes mL^−1^ in the toluene-degrading microcosms without Cd, 5.60 ± 0.39 log_10_ units in the presence of 4.4 µM Cd, and 4.32 ± 0.66 log_10_ units in the microcosms with 440 µM Cd.

Four phylogenetic groups were found to be significantly more abundant in the toluene-degrading microcosms with Cd co-contamination. These groups were the *Ralstonia* and *Cupriavidus* group, *Azoarcus tolulyticus*, *Rhodococcus* spp. and *Nitrosospira* spp. The *Ralstonia* and *Cupriavidus* group was the most abundant phylogenetic group measured in the microcosms with 4.4 µM, 44 µM, and 440 µM of Cd co-contamination. This group was also fairly abundant in the toluene-degrading control microcosms (6.56 ± 0.05 log_10_ units of 16S rRNA gene mL^−1^) but was more than one order of magnitude higher in the microcosms with 440 µM of Cd (7.65 ± 0.09 log_10_ units). The *Nitrosospira* spp. group was the most abundant measured phylogenetic group in the 0.44 µM Cd microcosms with 6.84 ± 0.26 log_10_ 16S rRNA genes mL^−1^, but this is only 0.67 log_10_ units higher than the no metal controls. It is worth mentioning that the *Nitrosospira* are ammonium oxidizing bacteria (Norton et al. 2008) and were likely using the ammonium amended with the mineral medium as an electron donor and not the hydrocarbon. The *Azoarcus tolulyticus* was measured below detection limits in the toluene control, but in the 0.44 µM Cd co-contaminated microcosms it was 6.09 ± 0.17 log_10_ units of 16S rRNA gene mL^−1^ and was 6.98 ± 0.46 log_10_ units in the microcosms with 440 µM Cd. The *Rhodococcus* spp. similarly showed higher abundances with successively higher Cd co-contamination. In the toluene-degrading control, it was 5.54 ± 0.07 log_10_ units of 16S rRNA gene mL^−1^ but was 6.90 ± 0.19 log_10_ units in the microcosms with 440 µM Cd.

The toluene-degrading microcosms co-contaminated with Pb showed some similarities to those contaminated with Cd at low concentrations. The 0.24 µM Pb co-contaminated microcosms had 15 phylogenetic groups with significantly lower abundances compared to the metals-free control. This includes nine of the ten groups that were also significantly lower in the 0.44 µM Cd co-contaminated microcosms, including the three most abundant groups in the toluene-degrading control (*Sporosarcina* and *Paenisporosarcina* spp., *Lysobacter spongiicola*, and *Aurantisolimonas* spp.). Only one group, the *Rhodococcus* spp., was significantly higher in the 0.24 µM Pb co-contaminated microcosms. Similar to the Cd-contaminated microcosms, the *Rhodococcus* spp. increased with higher amounts of Pb, reaching 7.28 ± 0.12 log_10_ units of 16S rRNA gene mL^−1^ in the microcosms with 240 µM of Pb. *Nitrosospira* spp. were also enriched in the Pb co-contaminated microcosms when 2.4 µM or more Pb was present. *Nitrosospira* increased to 7.45 ± 0.17 log_10_ units of 16S rRNA gene mL^−1^ in the 240 µM of Pb.

Otherwise, the Pb and Cd co-contaminated toluene degrading communities differed. At the lowest levels of Pb co-contamination, a reduction of several bacteria groups was observed indicating toxicity from the Pb. At the levels higher than 2.4 µM of Pb, however, the number of groups that were significantly lower than the control diminished. At 240 µM Pb co-contamination, only two groups were statistically significantly lower, and in both cases, the decrease was minimal (less than 0.4 log_10_ units). Eight groups were statistically significantly higher, including the *Lysobacter spongiicola* and the *Noviherbaspirillum* and *Herbaspirilllum* groups which were amongst the negatively impacted genera at 0.24 µM of Pb. These results may be due to the use of Pb-acetate salt for the amendment of Pb. In the microcosms with high levels of Pb co-contamination, the amount of acetate added at the beginning of the experiments (0.24 mM) likely had a priming impact and developed high abundances of the bacterial community. The amount of toluene added (eight rounds of 0.19 mM) may have been insufficient to completely discern the toluene degrading community above this acetate degrading community. Additionally, the acetate could have reduced the toxicity affects in the high Pb-amended microcosms. Another key difference between the Cd and Pb amendments were that the *Ralstonia* and *Cupriavidus* group the and *Azoarcus tolulyticus* were heavily enriched with Cd co-contamination but not with Pb. This suggests strongly that these two groups had a distinctive advantage under Cd co-contamination compared to uncontaminated or Pb contaminated systems.

In the benzene degrading communities, the largest bacterial groups in the no-metals added control were two different groups of *Pseudomonas spp*. and, similar to toluene, the *Lysobacter spongiicola*. A total of five assays were designed to target unique sequences within the *Pseudomonas* genus which were recovered from the high-throughput 16S rRNA gene amplicon sequencing (phylogenetic analysis located in Supplementary Information). The two groups which were of high abundance here were closely similar (based on 16S rRNA gene identity) to *Pseudomonas* st. P11-9 (Wu and Sun 2020) (the second of the five assays), and *Pseudomonas putida* st. HB3S-20 (LiRong et al. 2018) (the fourth of the five assays). In the cultures co-contaminated with 440 µM of Cd, 16 phylogenetic groups had statistically significantly lower abundances than the no metal control. However, the number of groups lower at 0.44–44 µM Cd was much less than exhibited in the toluene degrading cultures. For example, while *Lysobacter spongiicola* had lower abundances in the toluene degrading cultures at all levels of Cd co-contamination, benzene degradation cultures became more dominant with 4.4 µM and 44 µM of Cd. Indeed, it was the most dominant community member in the benzene degrading cultures with 4.4 µM Cd co-contamination. *Lysobacter spongiicola* was significantly lower at 440 µM of Cd. The two groups of *Pseudomonas* also showed no population decrease, except at the highest level of Cd co-contamination. Overall, the abundances of the five different *Pseudomonas spp.* were 1–2 orders of magnitude higher in the benzene degrading cultures than in the toluene degrading cultures with co-contamination of Cd up to 44 µM.

The most abundant bacterial group in the benzene degrading cultures differed at each concentration of Cd. At 0.44 µM of Cd, the group of *Sporosarcina* and *Paenisporosarcina spp*. was the most dominant with 7.03 ± 0.08 log_10_ units of 16S rRNA gene mL^−1^. At 4.4 µM Cd, *Lysobacter spongiicola* was the most prominent community member at 7.50 ± 0.03 log10 units of 16S rRNA gene mL^−1^. At 44 µM, the *Nitrosospira spp.,* which also showed a slight (though statistically significant) increase in the toluene-degrading microcosms at intermediate Cd concentrations, had an immense abundance at 7.79 ± 0.45 log_10_ units of 16S rRNA gene mL^−1^. At 440 µM Cd, the most dominant group was the *Ralstonia* and *Cupriavidus* group at 7.87 ± 0.23 log_10_ units of 16S rRNA gene mL^−1^. The second most abundant group was the *Arthrobacter* and *Pseudoarthrobacter* at 6.99 ± 0.39 log_10_ units of 16S rRNA gene mL^−1^, though this was not significantly higher than at the lower levels of Cd co-contamination. These results suggest that compared to toluene-degrading cultures, the benzene-degrading cultures are less impacted by Cd co-contamination up to 44 µM. However, at high levels of Cd co-contamination, the *Ralstonia* and *Cupriavidus* have distinctive advantages with both toluene and benzene degradation.

The benzene degrading cultures were also impacted by Pb co-contamination. The most dominant *Pseudomonas* group in the benzene-degrading no metals control microcosms was lowered from 7.24 ± 0.53 log_10_ units of 16S rRNA gene mL^−1^ to 5.52 ± 0.51 log_10_ units with 240 µM Pb. This *Pseudomonas* was also lower in two triplicate microcosms with 24 µM of Pb. However, the Pb concentrations did not impact the other groups of *Pseudomonas* and *Lysobacter spongiicola*. Though the *Ralstonia* and *Cupriavidus* were not favored in the toluene-degrading cultures with Pb co-contamination, they were significantly higher in the benzene-degrading cultures with Pb co-contamination. With 240 µM Pb, the *Ralstonia* and *Cupriavidus* group was measured at 7.85 ± 0.32 log_10_ units of 16S rRNA gene mL^−1^. The *Nitrosospira* spp. was also increased with Pb co-contamination and was 7.71 ± 0.32 log_10_ units of 16S rRNA gene mL^−1^ with 240 µM Pb. In addition to the *Pseudomonas* group aforementioned, a *Bacillus sp*. group and *Arenimonas spp.* were measured to be significantly lower at 240 µM Pb. At the intermediate concentration of 2.4 µM Pb, an uncultured *Sphingobacteriales* group was present at 7.05 ± 0.09 log_10_ units of 16S rRNA gene mL^−1^, a complete order of magnitude higher than at any other condition. Still, at this concentration of Pb, two measured groups of *Pseudomonas spp.*, *Lysobacter spongiicola,* the *Ralstonia* and *Cupriavidus* group were higher in concentration.

**Fig. 4:**
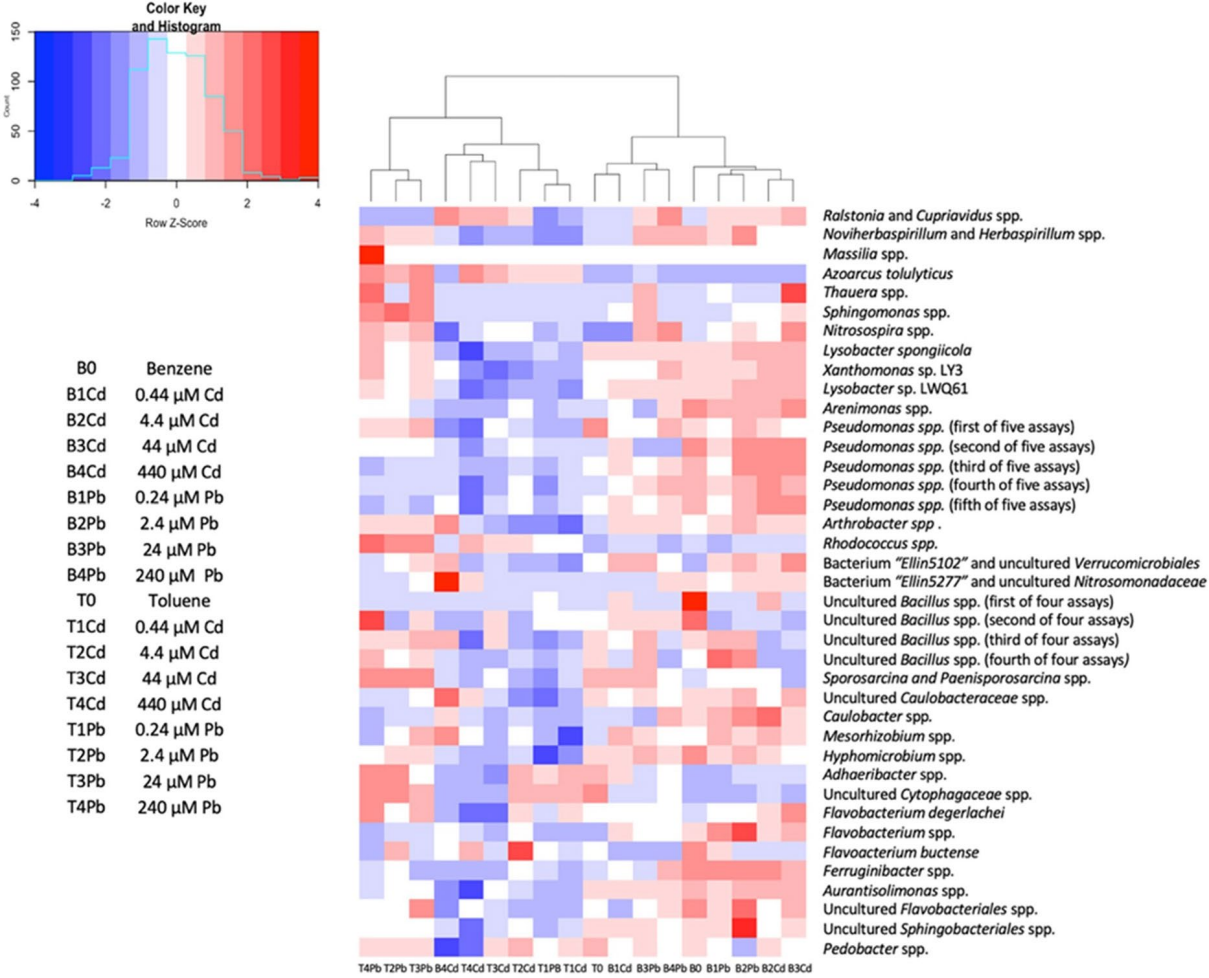
Dendrogram and heatmap (correlation distance) analysis of the 39 Bacteria in an OTU level most abundant and unique for the co-contaminants’ type and concentration. The scale bar and heatmap indicates the log-unit increase or decrease for each OTU from 16S rRNA in the sample, derived from qPCR analysis, compared with its mean. Dark blue corresponds to low expression, dark red corresponds to high expression

A dendrogram and heatmap analysis of the qPCR results is shown in Fig. 4. The heatmap data is centered on the average for bacterial groups, thus showing how each microbial community either was underrepresented or overrepresented in that condition compared to the average across all conditions. The dendrogram shows that the microbial community amended with benzene and 440 µM Cd differed from the other benzene-amended microcosms. This agrees with the NMDS analysis (based on the 16S rRNA amplicon sequencing) shown in Fig. 3. The benzene degrading microcosms with high amounts of Cd produced shifts in the microbial community most similar to that observed in the toluene degrading microcosms with that level of Cd. In the toluene degrading microcosms, the lowest levels of Cd and Pb provided a similar shift in the communities compared to the metals-free control. However, higher levels of Cd and Pb produced distinctive shifts from each other. This again agrees with the NMDS analysis based on amplicon sequencing.

## Discussion

In our study, the soil collected from a stormwater runoff basin was found to provide fastidious degradation of benzene and toluene. Our experiments used mostly liquid microcosms. In a soil environment, however, sorption of toluene and benzene to solids most likely leads to lower hydrocarbon bioavailability resulting in lower degradation rates. Metal sorption and speciation also can be complex. The degradation rates and lag times in our experiments did not significantly decrease due to Cd or Pb co-contamination. Toluene had a higher degradation rate than benzene, which has often been observed in other studies (Carvajal et al. 2018; Chang et al. 1993; Collins and Daugulis 1999; de Nardi et al. 2007). Toluene is readily biodegradable under aerobic conditions if it is the sole carbon and energy source (Carvajal et al. 2018). Lin et al. (2007) and Amor et al. (2001) reported the inhibitory effects of heavy metals on BTEX degraders, finding that effects were heavy metal concentration dependent. In our study, the lack of inhibition from the metals was unexpected. Heavy metals can prevent the biodegradation of contaminants due to disrupting enzyme activities directly involved in biodegradation (Amor et al. 2001). In bioremediation, the toxicity of metals on the degradation of organic pollutants at contaminated sites has been well-documented (Sandrin and Maier 2003). Research with pure cultures has confirmed the toxicity effects of heavy metals on commonly known benzene and toluene degrading cultures. For example, a concentration of 330 µM of Cd reduced toluene degradation by more than half for both a *Bacillus* and *Pseudomonas* (Amor et al. 2001).

In our study, 39 microbial groups were measured with qPCR after several rounds of benzene or toluene additions under the range of concentrations of Cd and Pb co-contamination. Ten of these groups were more than 10^7^ copies of 16S rRNA genes mL^−1^, and another 17 were more pronounced than 10^6^ copies of 16S rRNA genes mL^−1^, in at least one condition. It suggests that this soil contained numerous and diverse toluene and benzene degraders. The diversity of putative hydrocarbon degraders in this one soil and ability to degrade benzene and toluene under various metal conditions agrees with the biological insurance theory that states that biodiversity buffers ecosystem functioning against the disruptive effects of environmental fluctuations (Yachi and Loreau 1999). From an ecological perspective, several theoretical and empirical arguments were contributed on why a diversity of species responses should be expected to buffer ecosystems against environmental fluctuations and thereby enhance the stability of ecosystem functioning. Biodiversity is insuring against extremes in ecosystem functioning and can enhance ecosystem functioning in fluctuating environments (MacArthur 1955; McNaughton 1977; Patten 1975). Bacteria can adapt to heavy metal pollution. A sensitive species can adapt to heavy metals but still be less competitive than those species, which can already resist the contaminant. This competition is incredibly intense in the soil system (Bååth et al. 1998). Bååth et al. (1998) demonstrated that the tolerance of microbial communities was the highest, where the concentration of metals was the highest.

In all conditions in our study, the benzene degrading microcosms had fewer major bacteria present than toluene degrading microcosms. It is not surprising considering the smaller breadth of known benzene degraders compared to toluene degraders (Amor et al. 2001; Lin et al. 2007; Kozdrój and van Elsas 2001). Similar to our findings, Lin et al. (2007) and Amor et al. (2001) found samples generated distinctly different community profiles when heavy metals were present together with BTEX and MTBE. The presence of heavy metals decreased bacterial community diversity and a difference in the overall community structure.

In previous research, most of the genera measured have been implicated as toluene and/or benzene degraders. Exceptions to this include *Nitrosospira,* an ammonium oxidizing bacteria that most likely utilize the ammonium added with the mineral media to all microcosms (Norton et al. 2008). The *Nitrosospira* were found to tolerate heavy metals, which is not surprising as previous research has found this genus to tolerate heavy metal environments (Luo et al. 2019; Mertoglu et al. 2008). Another exception is the *Aurantisolimonas* genus, which only recently contained an isolated and named strain (Liu et al. 2018). This microbe was one of the more abundant members in the toluene and benzene control (no metals added) microcosms and was negatively impacted by high amounts of Cd co-contamination. Further research is required to determine if this genus of bacteria has strains capable of toluene or benzene biodegradation.

The bacteria genera detected, however, are typically found in benzene and toluene degrading cultures. This includes the *Pseudomonas*, *Azoarcus*, and *Rhodococcus,* though other degraders such as *Aminobacter* and *Burkholderia* were not found in our experiments (Chang et al. 1993; Genovese et al. 2008; Hendrickx et al. 2006; Lhotský et al. 2017; Sperfeld et al. 2018; Parales et al. 2000). The *Cupriavidus* and *Ralstonia*–like genera were found to dominate in the Cd contaminated toluene degrading microcosms and in the benzene degrading microcosms with either Pb or Cd. However, it was at a lower abundance in most of the high Pb-toluene, indicating that it was not as competitive under those conditions as the other genera present and benzene samples co-contaminated with Cd or Pb when compared to the control samples. Several strains within this group have been isolated and found to degrade aromatic hydrocarbons (Alviz-Gazitua et al. 2022; Morya and Salvachúa 2020; Witzig et al. 2006). For example, *Cupriavidus metallidurans* st. CH34 has degraded benzene and toluene with Hg and Cd co-contamination (Alviz-Gazitua et al. 2022). In that study, the minimum inhibitory concentration of Cd was found to be 0.8 mM, which is greater than the concentrations we tested.

The number of *Azoarcus tolulyticus* increased significantly in our study in the toluene samples co-contaminated with Cd. *Azoarcus tolulyticus* originates from soils that is associated with nitrogen-fixing (Chauhan et al. 2015) and denitrification (Zhou et al. 1995), and it has previously been found degrade aromatic compounds like toluene or phenol (Song et al. 1999). *Azoarcus* was present in microcosms with a high abundance of *Nitrosomonas* spp., which oxidizes ammonia to nitrate. We did not measure nitrate, but it is possible that *Azoarcus* in our cultures could also have been denitrifying and using the nitrate as an electron acceptor. Similarly, denitrification could have been a factor with other microbial changes we found since several general, including *Pseudomonas* and *Lysobacter* spp., are often capable of denitrification (Carvajal et al. 2018). Alterations by the microbial community to our amended metals were also not determined but could also add complexity within co-contaminated environments. For example, *Pseudomonas aeruginosa* can produce metal-chelating siderophores that improve metal bioavailability (Agnello et al. 2016).

Finding microbes that are resistant to heavy metal toxicity and can degrade benzene and toluene simultaneously is essential to remediation efforts in co-contaminated sites. In our work, a soil microbial community with diverse toluene and benzene degrading populations has shown the ability to degrade benzene (0.23 mM) or toluene (0.19 mM) under various concentrations of Cd and Pb. Further research is required to better understand the bioremediation potential for long-term co-contaminants at hazardous waste sites. However, this study suggests that either native populations or bioaugmentation cultures with diverse populations of degraders increases chances of success in co-contaminated environments.

## Supplementary Information

Below is the link to the electronic supplementary material.

Supplementary file1 (PDF 428 KB)

## References

[CR1] Agnello AC, Bagard M, van Hullebusch ED, Esposito G, Huguenot D (2016). Comparative bioremediation of heavy metals and petroleum hydrocarbons co-contaminated soil by natural attenuation, phytoremediation, bioaugmentation and bioaugmentation-assisted phytoremediation. Sci Total Environ.

[CR2] Alviz-Gazitua P, Duran RE, Millacura FA, Cardenas F, Rojas LA, Seeger M (2022). *Cupriavidus metallidurans* CH34 possesses aromatic catabolic versatility and degrades benzene in the presence of mercury and cadmium. Microorganisms.

[CR3] Amor L, Kennes C, Veiga MC (2001). Kinetics of inhibition in the biodegradation of monoaromatic hydrocarbons in presence of heavy metals. Biores Technol.

[CR4] Bååth E, Díaz-Raviña M, Frostegård A, Campbell CD (1998). Effect of metal-rich sludge amendments on the soil microbial community. Appl Environ Microbiol.

[CR5] Carvajal A, Akmirza I, Navia D, Perez R, Munoz R, Lebrero R (2018). Anoxic denitrification of BTEX: biodegradation kinetics and pollutant interactions. J Environ Manage.

[CR6] Chang M-K, Voice TC, Criddle CS (1993). Kinetics of competitive inhibition and cometabolism in the biodegradation of benzene, toluene, and p-xylene by two *Pseudomonas* isolates. Biotechnol Bioeng.

[CR7] Chauhan H, Bagyaraj DJ, Selvakumar G, Sundaram SP (2015). Novel plant growth promoting rhizobacteria-prospects and potential. Appl Soil Ecol.

[CR8] Collins LD, Daugulis AJ (1999). Benzene/toluene/p-xylene degradation, Part I. Solvent selection and toluene degradation in a two-phase partitioning bioreactor. Appl Microbiol Biotechnol.

[CR9] De Nardi IR, Zaiat M, Foresti E (2007). Kinetics of BTEX degradation in a packed-bed anaerobic reactor. Biodegradation.

[CR10] Genovese M, Denaro R, Capello S, Di Marco G, La Spada G, Guiliano L, Genovese L, Yakimov MM (2008). Bioremediation of benzene, toluene, ethylbenzene, xylenes-contaminated soil: a biopile pilot experiment. J Appl Microbiol.

[CR11] Hendrickx B (2006). Alternative primer sets for PCR detection of genotypes involved in bacterial aerobic BTEX degradation: distribution of the genes in BTEX degrading isolates and in subsurface soils of a BTEX contaminated industrial site. J Microbiol Methods.

[CR12] Kozdrój J, van Elsas JD (2001). Structural diversity of microbial communities in arable soils of a heavily industrialised area determined by PCR-DGGE fingerprinting and FAME profiling. Appl Soil Ecol.

[CR13] Kumar S, Stecher G, Li M, Knyaz C, Tamura K (2018). MEGA X: molecular evolutionary genetics analysis across computing platforms. Mol Biol Evol.

[CR14] Lhotský O (2017). Assessment of biodegradation potential at a site contaminated by a mixture of BTEX, chlorinated pollutants and pharmaceuticals using passive sampling methods—Case study. Sci Total Environ.

[CR15] Lin C-W, Lin H-C, Lai C-Y (2007). MTBE biodegradation and degrader microbial community dynamics in MTBE, BTEX, and heavy metal-contaminated water. Int Biodeterior Biodegrad.

[CR16] LiRong J, Peng W, Wei H (2018). Biocontrol effect on cotton Verticillium wilt and identification of endophytic bacterium HB3S-20 from cotton. J Henan Agric Sci.

[CR17] Liu M-J, Jin C-Z, Asem MD, Ju Y-J, Park D-J, Salam N, Xiao M, Li W-J, Kim C-J (2018). *Aurantisolimonas haloimpatiens* gen. nov., sp. nov., a bacterium isolated from soil. Int J Syst Evol Microbiol.

[CR18] Lookman R, Bastiaens L, Borremans B, Maesen M, Gemoets J, Diels L (2004). Batch-test study on the dechlorination of 1,1,1-trichloroethane in contaminated aquifer material by zero-valent iron. J Contam Hydrol.

[CR19] Lozano TM, McCutchan AL, Krzmarzick MJ (2019). Hydraulic fracturing fluid compositions induce differential enrichment of soil bacterial communities. Environ Eng Sci.

[CR20] Luo J, Liu Y, Tao Q, Hou Q, Wu K, Song Y, Liu Y, Guo X, Li J, Hashmi MLuR, Liang Y, Li T (2019). Successive phytoextraction alters ammonia oxidation and associated microbial communities in heavy metal contaminated agricultural soils. Sci Total Environ.

[CR21] MacArthur RH (1955). Fluctuations of animal populations and a measure of community stability. Ecology.

[CR22] Madeira CL, Jog KV, Vanover ET, Brooks MD, Taylor DK, Sierra-Alvarez R, Waidner LA, Spain JC, Krzmarzick MJ, Field JA (2019). Microbial enrichment culture responsible for the complete oxidative biodegradation of 3-amino-1,2,4-triazol-5-one (ATO), the reduced daughter product of the insensitive munitions compound 3-nitro-1,2,4-triazol-5-one (NTO). Environ Sci Technol.

[CR23] Madrid F, Rubio-Bellido M, Villaverde J, Tejada M, Morillo E (2016). Natural attenuation of fluorene and pyrene in contaminated soils and assisted with hydroxypropyl-β-cyclodextrin. Effect of co-contamination. Sci Total Environ.

[CR24] McNaughton SJ (1977). Diversity and stability of ecological communities: a comment on the role of empiricism in ecology. Am Nat.

[CR25] Mertoglu B, Semerci N, Guler N, Calli B, Cecen F, Saatci AM (2008). Monitoring of population shifts in an enriched nitrifying system under gradually increased cadmium loading. J Hazard Mater.

[CR26] Morya R, Salvachúa D (2020). *Burkholderia*: an untapped but promising bacterial genus for the conversion of aromatic compounds. Trends Biotechnol.

[CR27] Norton JM, Klotz MG, Stein LY, Arp DJ, Bottomley PJ, Chain PSG, Hauser LJ, Land ML, Larmer FW, Shin MW, Starkenburg SR (2008). Complete genome sequence of *Nitrosospira multiformis*, an ammonia-oxidizing bacterium from the soil environment. Appl Environ Microbiol.

[CR28] Parales RE, Ditty JL, Harwood CS (2000). Toluene-degrading bacteria are chemotactic towards the environmental pollutants' benzene, toluene, and trichloroethylene. Appl Environ Microbiol.

[CR29] Patten BC (1975). Ecosystem linearization: an evolutionary design problem. Am Nat.

[CR30] Roane TM, Josephson KL, Pepper IL (2001). Dual-bioaugmentation strategy to enhance remediation of cocontaminated soil. Appl Environ Microbiol.

[CR31] Sandrin TR, Maier RM (2003). Impact of metals on the biodegradation of organic pollutants. Environ Health Perspect.

[CR32] Schwarz A, Adetutu EM, Juhasz AL, Aburto-Medina A, Ball AS, Shahsavari E (2019). Response of the fungal community to chronic petrogenic contamination in surface and subsurface soils. Geoderma.

[CR33] Shelton DR, Tiedje JM (1984). General method for determining anaerobic biodegradation potential. Appl Environ Microbiol.

[CR34] Song B, Häggblom MM, Zhou J, Tiedje JM, Pallerino NJ (1999). Taxonomic characterization of denitrifying bacteria that degrade aromatic compounds and description of *Azoarcus toluvorans* sp. nov. and *Azoarcus toluclasticus* sp. nov. Int J Syst Evol Microbiol.

[CR35] Speight JG (2014). The chemistry and technology of petroleum.

[CR36] Sperfeld M, Rauschenbach C, Diekert G, Studenik S (2018). Microbial community of a gasworks aquifer and identification of nitrate-reducing *Azoarcus* and *Georgfuchsia* as key players in BTEX degradation. Water Res.

[CR37] U.S. Environmental Protection Agency (US EPA) (2020) Current NPL Updates: New Proposed NPL Sites and New NPL Sites. https://www.epa.gov/superfund/current-npl-updates-new-proposed-npl-sites-and-new-npl-sites

[CR38] Vaezihir A, Zare M, Raeisi E, Molson J, Barker J (2012). Field-scale modeling of benzene, toluene, ethylbenzene, and xylenes (BTEX) released from multiple source zones. Bioremediat J.

[CR39] Weelink SAB, van Eekert MHA, Stams AJM (2010). Degradation of BTEX by anaerobic bacteria: physiology and application. Rev Environ Sci Biotechnol.

[CR40] Witzig R, Junca H, Hecht H-J, Pieper DH (2006). Assessment of toluene/biphenyl dioxygenase gene diversity in benzene-polluted soils: links between benzene biodegradation and genes similar to those encoding isopropylbenzene dioxygenases. Appl Environ Microbiol.

[CR41] Wu, Q., Sun, L. (2020). Heavy metals resistant bacteria isolated from the sediments of Baiyangdia Lake. Unpublished. 16S rRNA gene sequence of “*Pseudomonas* sp. strain P11-9” submitted to NCBI GenBank, Accession # MT255077. https://www.ncbi.nlm.nih.gov/nuccore/MT255077

[CR42] Yachi S, Loreau M (1999). Biodiversity and ecosystem productivity in a fluctuating environment: the insurance hypothesis. Proc Natl Acad Sci USA.

[CR43] Zehnder AJB, Wuhrmann K (1977). Physiology of a *Methanobacterium* strain AZ. Arch Microbiol.

[CR44] Zhou J, Fries MR, Chee-Sanford JC, Tiedje JM (1995). phylogenetic analyses of a new group of denitrifiers capable of anaerobic growth on toluene and description of *Azoarcus tolulyticus* sp. nov. Int J Syst Evol Microbiol.

[CR45] Zukauskaite A, Jakubauskaite V, Belous O, Ambrazaitiene D, Stasiskiene Z (2008). Impact of heavy metals on the oil products biodegradation process. Waste Manage Res.

