# Supplementary material for: Lead or cadmium co-contamination alters benzene and toluene degrading bacterial communities
Source: Biodegradation. 2023 Feb 25;34(4):357–69. doi: 10.1007/s10532-023-10021-w (PMC10191895; doi:10.1007/s10532-023-10021-w)
Supplement: Supplementary file 1 — Supplementary file1 (PDF 428 KB) [file 10532_2023_10021_MOESM1_ESM.pdf]

Supplementary Information
For

Lead or Cadmium Co-Contamination Alters Benzene and Toluene Degrading Bacterial Communities

Aniko Konya[1], Brice A. Fiddler[2], Olivia Bunch[2], Kendra Z. Hess[2], Cade Ferguson[2], and Mark J. Krzmarzick [1,2]

[1] Environmental Science Graduate Program, Oklahoma State University, Stillwater, OK, USA

[2] School of Civil and Environmental Engineering, Oklahoma State University, Stillwater, OK, USA

Corresponding Author: Mark J. Krzmarzick; e-mail: mark.krzmarzick@okstate.edu

Table S1 The composition of the mineral medium (Shelton & Tiedje, 1984).

**Compound Name**	**Concentration**
NaCl	1,000 mg/L
$MgCl_2*6H_2O$	500 mg/L
KH_2PO_4	200 mg/L
NH_4Cl	300 mg/L
KCl	300 mg/L
$CaCl_2$	15 mg/L
Trace Elements A	1 mL/L
Trace Elements B	1 mL/L
Yeast Extract	10 mg/L
**Trace Elements Solution A** (Zhender & Wuhrmann, 1977)	
**Compound**	**Concentration**
$FeCl_2*4H_2O$	1.5 g/L
$CoCl_2*6H_2O$	0.19 g/L
$MnCl_2*4H_2O$	0.1 g/L
$ZnCl_2$	70 mg/L
H_3BO_3	6 mg/L
$Na2MoO_4$	36 mg/L
$NiCl_2*6H_2O$	24 mg/L
$CuCl_2*2H_2O$	2 mg/L
25% HCl	10 ml/L
**Trace Elements Solution B** (Zhender & Wuhrmann, 1977)	
**Compound**	**Concentration**
$Na_2WO_4*2H_2O$	8 mg/L
NaOH	0.5 g/L
$Na_2SeO_3*5H_2O$	6 mg/L

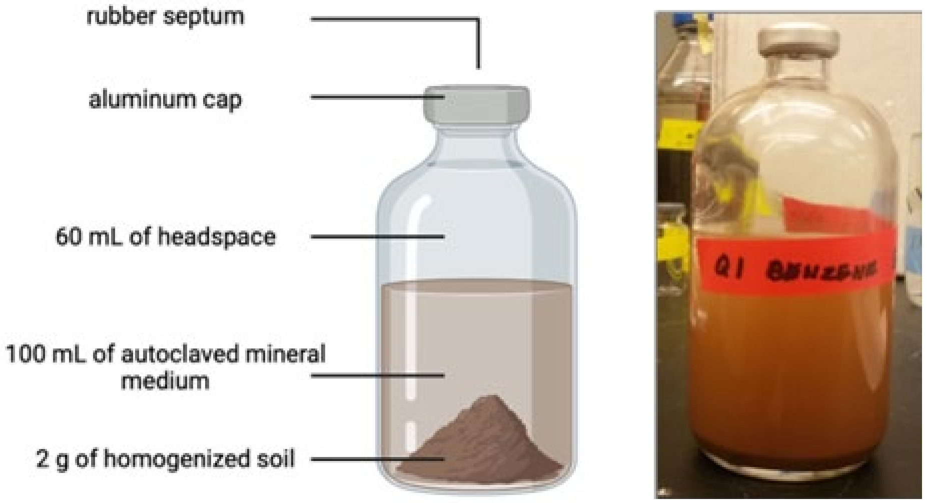

Figure S1. Schematic of the serum bottles used for the microcosms on the left and an image of an original microcosm on the right.

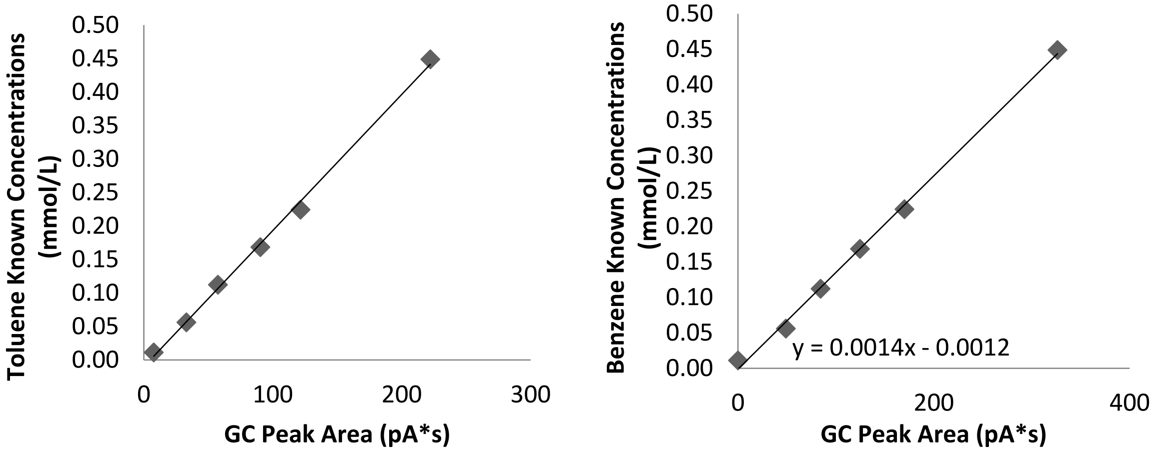

Figure S2. Standard curves for toluene and benzene analysis using GC-FID. This quantification limit was found to be 0.009 mM for toluene and 0.011 for benzene. Microcosms were initially amended with 0.19 mM of toluene or 0.23 mM of benzene. Standards covering the range of concentrations found in the microcosms were linear with GC response.

Table S2. QPCR assays used and developed for quantification of phylogenetic groups in this study. Phylogenetic analysis is shown in Figure S3, primers and amplicon size for each assay are shown in Table S3, qPCR assay parameters are shown in Table S4, and quantification results are shown in Tables S5-S8.

Assay #	OTUs targeted	Phylogenetic Classification and notes
1	72, 325, 5847	*Ralstonia* and *Cupriavidus* spp.
2	161	*Noviherbaspirillum* and *Herbaspirillum* spp.
3	2815	*Massilia* spp.
4	745	*Azoarcus tolulyticus*
5	22	*Thauera* spp.
6	176	*Sphingomonas* spp.
7	31, 356	*Nitrosospira* spp.
8	1817, 6399	*Lysobacter spongiicola*
9	121	*Xanthomonas* sp. LY3
10	21	*Lysobacter* sp. LWQ61
11	3504	*Arenimonas* spp.
12	110	*Pseudomonas spp.* (first of five assays)
13	1, 4554, 7128	*Pseudomonas spp.* (second of five assays)
14	6205	*Pseudomonas spp.* (third of five assays)
15	6872	*Pseudomonas spp.* (fourth of five assays)
16	6940	*Pseudomonas spp.* (fifth of five assays)
17	3, 3624, 4661, 4733	*Arthrobacter* and *Pseudarthrobacter spp.*
18	9, 52, 2918	*Rhodococcus spp.*
19	254	Bacterium *"Ellin5102"* and uncultured *Verrucomicrobiales*
20	141	Bacterium *"Ellin5277"* and uncultured *Nitrosomonadaceae*
21	16, 5712	Uncultured *Bacillus* spp. (first of four assays)
22	11	Uncultured *Bacillus* spp. (second of four assays)
23	5793	Uncultured *Bacillus* spp. (third of four assays)
24	47, 4705	Uncultured *Bacillus* spp. (fourth of four assays*)*
25	12, 13, 1663, 1888	*Sporosarcina and Paenisporosarcina* spp.
26	147	*Caulobacteraceae* spp.
27	6151	*Caulobacter* spp.
28	89	*Mesorhizobium* spp.
29	391	*Hyphomicrobium* spp.
30	330	*Adhaeribacter* spp.
31	3353	*Cytophagaceae* spp.
32	36	*Flavobacterium degerlachei*
33	117	*Flavobacterium* spp.
34	123	*Flavoacterium buctense*
35	3740	*Ferruginibacter* spp.
36	7	*Aurantisolimonas* spp.
37	90	Uncultured *Flavobacteriales* spp.
38	61	Uncultured *Sphingobacteriales* spp.
39	4517, 5181	*Pedobacter* spp.

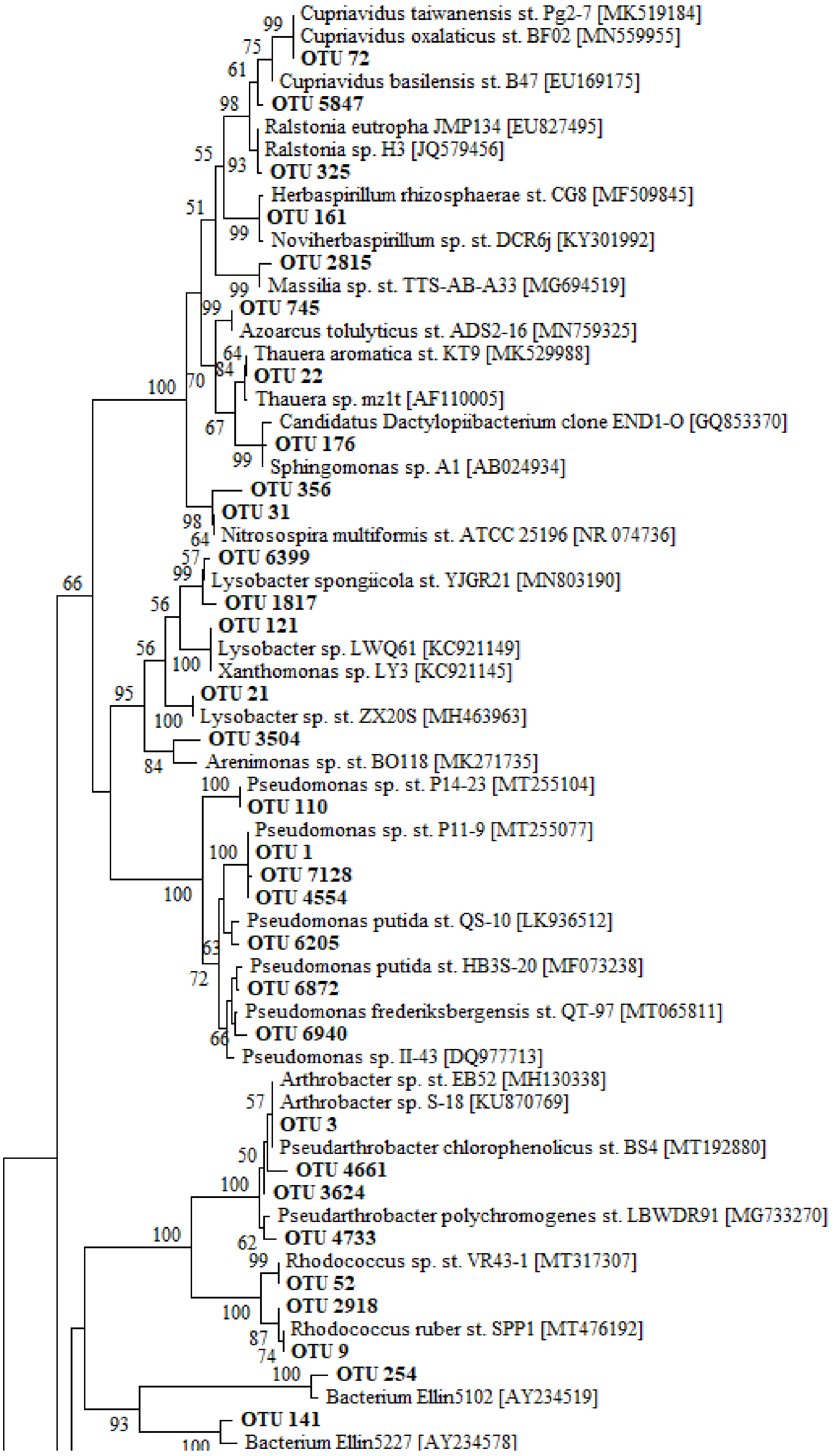

Cupriavidus taiwanensis st. Pg2-7 [MK519184]
Cupriavidus oxalaticus st. BF02 [MN559955]
OTU 72
Cupriavidus basilensis st. B47 [EU169175]
OTU 5847
Ralstonia eutropha JMP134 [EU827495]
Ralstonia sp. H3 [JQ579456]
OTU 325
Herbaspirillum rhizosphaerae st. CG8 [MF509845]
OTU 161
Noviherbaspirillum sp. st. DCR6j [KY301992]
OTU 2815
Massilia sp. st. TTS-AB-A33 [MG694519]
OTU 745
Azoarcus tolulyticus st. ADS2-16 [MN759325]
Thauera aromatica st. KT9 [MK529988]
OTU 22
Thauera sp. mz1t [AF110005]
Candidatus Dactylopiibacterium clone END1-O [GQ853370]
OTU 176
Sphingomonas sp. A1 [AB024934]
OTU 356
OTU 31
Nitrosospira multiformis st. ATCC 25196 [NR 074736]
OTU 6399
Lysobacter spongiicola st. YJGR21 [MN803190]
OTU 1817
OTU 121
Lysobacter sp. LWQ61 [KC921149]
Xanthomonas sp. LY3 [KC921145]
OTU 21
Lysobacter sp. st. ZX20S [MH463963]
OTU 3504
Arenimonas sp. st. BO118 [MK271735]
Pseudomonas sp. st. P14-23 [MT255104]
OTU 110
Pseudomonas sp. st. P11-9 [MT255077]
OTU 1
OTU 7128
OTU 4554
Pseudomonas putida st. QS-10 [LK936512]
OTU 6205
Pseudomonas putida st. HB3S-20 [MF073238]
OTU 6872
Pseudomonas frederiksbergensis st. QT-97 [MT065811]
OTU 6940
Pseudomonas sp. II-43 [DQ977713]
Arthrobacter sp. st. EB52 [MH130338]
Arthrobacter sp. S-18 [KU870769]
OTU 3
Pseudarthrobacter chlorophenolicus st. BS4 [MT192880]
OTU 4661
OTU 3624
Pseudarthrobacter polychromogenes st. LBWDR91 [MG733270]
OTU 4733
Rhodococcus sp. st. VR43-1 [MT317307]
OTU 52
OTU 2918
Rhodococcus ruber st. SPP1 [MT476192]
OTU 9
OTU 254
Bacterium Ellin5102 [AY234519]
OTU 141
Bacterium Ellin5227 [AY234578]

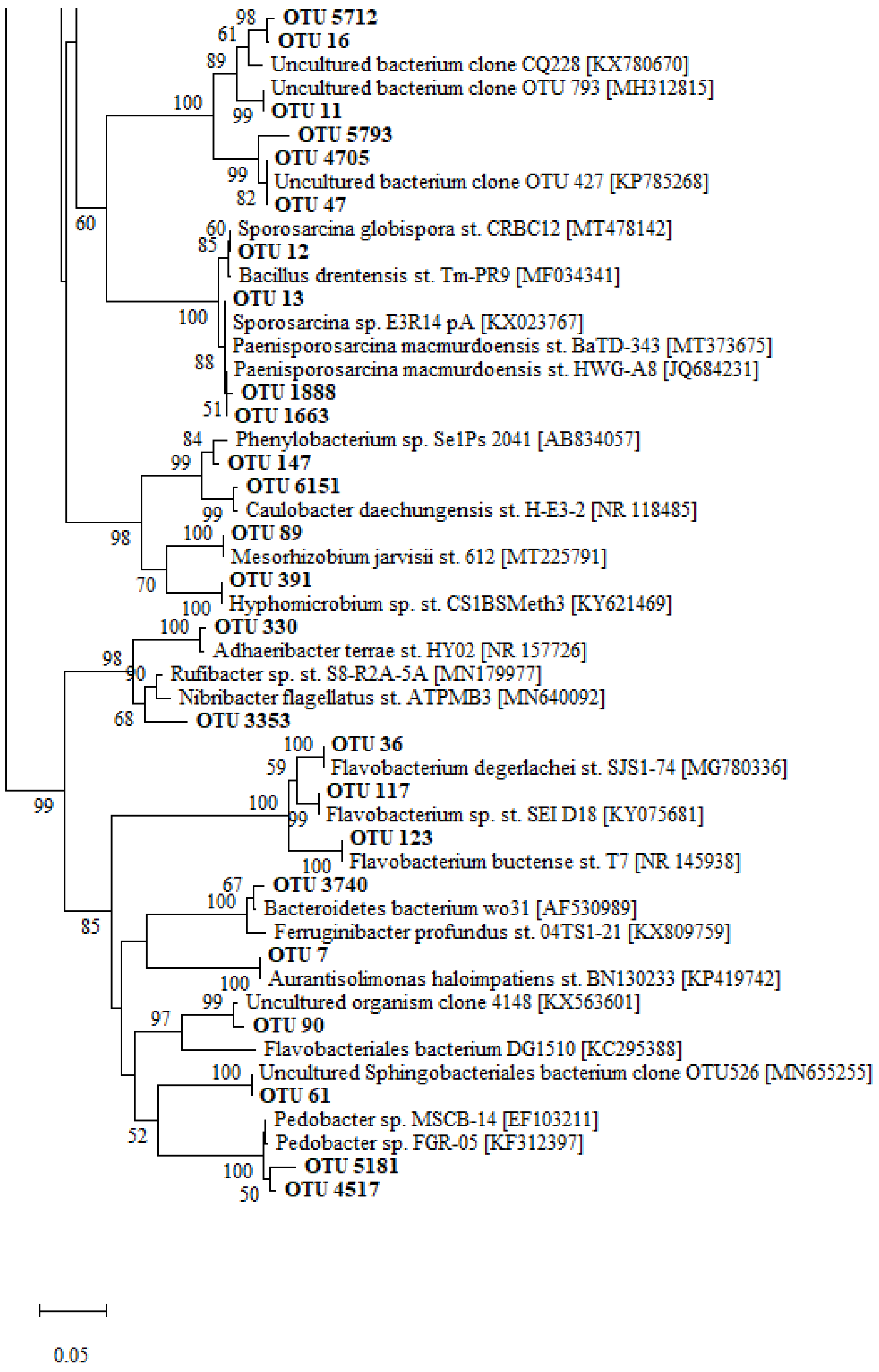

Figure S3. Phylogenetic analysis of the OTUs targeted for qPCR assays. A total of 293 bps were in the final dataset. The analysis was conducted in MEGA X (Kumar et al. 2018) with an alignment using MUSCLE (Edgar 2004). Tree configuration was developed with neighbor-joining method (Saitou and Nei, 1987) for evolutionary history and Maximum Composite Likelihood method (Tamura et al., 2004) for evolutionary distances. Bootstrap analysis (1000 replicates) was used for statistical tests (Felsenstein 1985); percentage of replicate trees in which the taxa clustered are shown next to the branches.

Table S3 The primers used in the qPCR assays. Primers were designed for this study, except where otherwise noted in the footnotes.

Assay	Forward Primer	Reverse Primer	Amplicon Size (bp)
1[a]	5’-GCATACGACCTGAGGGTGAAA-3’	5’-TGTGGCTGATCGTCCTCTCA-3’	139
2[a]	5’- CCCTGGAGTGGGGGATAACT -3’	5’- AGCATGAGGTCTTGCGATCC -3’	87
3	5'- ACCTGGGAATTGCGATGGAG-3'	5'- CGCATTTCACTGCTACCACG-3'	81
4	5'- TTAACCTGGGAACTGCGCTT -3'	5'- CGCATTTCACTGCCTACACGT-3'	83
5	5'- TCAACCTGGGAACTGCGTTT-3'	5'- CCATCGGTGTTCCTCCTGAT-3'	107
6	5'- TTAACCTGGGAACTGCGCTT-3'	5'- CGCATTTCACTGCTACACCA-3'	82
7[b]	5’-GAAACTGCCGGTGACAAACC-3’	5’-CTTCTGGTGGAACCCACTCC-3’	282
8	5'- GTAGCGGAATTCCCGGTGTA-3'	5'- GTCAGTGCTGGTCCAGGTAG-3'	87
9	5'- ATGGCATTGGATACTGGCGG-3'	5'- CGGATGTTCCTCCCGATCTC-3'	93
10	5'- AGTCTGATGTGAAAGCCCCG- 3'	5'- GAATTCCGCCACCCTCTACC-3'	84
11	5'- TAGGCGGTTTTGTTAAGTCTGTC-3'	5'- CTCTAGCTCGCCAGTATTCCATT-3'	78
12	5'- GGGAACTGCATCCAAAACTACT-3'	5'- CCTCAGTGTCAGTATTAGTCCAGG-3'	136
13	5'- CGCGTAGGTGGTTGGTTAAG-3'	5'- TTCGCCACTGGTGTTCCTTC-3'	152
14	5'- GTTAAGTTGAATGTGAAAGCCCCG-3'	5'- TACACAGGAATTCCACCACCTCTAC-3'	94
15	5'- CGCGTTAGGTGGTTCGTTAAG-3'	5'- TTCGCCACTGGTGTTCCTTC-3'	154
16	5'- CAACCTGGGAACTGCATTCAA-3'	5'- TTCGCACTGTGTTCCTTCCT-3'	106
17	5'- GAAAGTCCGGGGCTCAACTCC-3'	5'- CTCAGCGTCAGTTAATGCCCAG-3'	155
18	5'- GTCGCGTCGTCTGTGAAAAC-3'	5'- GGAATTCCAGTCTCCCCTGC-3'	89
19	5'- AGGTGGTGAGGTAAGTCGGA -3'	5'- TGCCTCAGTGTCAGGTGTTG -3'	181
20	5'- CAACTCAGAGTCTGCAACGG -3'	5'- AGTTATGGTCCAGTGAGCCG -3'	132
21	5'- GGCGGTTTTCTAAGTCTGGG -3'	5'- TTTCGCCACTGGTGTTCCTC -3'	147
22	5'- GGCGGTTTACCAAGTCTGGA -3'	5'- TTTCGCCACTGGTGTTCCTC -3'	147
23	5'- GGCGGTTCCTTAAGTTTGGG -3'	5'- TTTCGCCACTGGTGTTCCTC -3'	147
24	5'- GGCGGTTCCTTAAGTTTGGA -3'	5'- TTTCGCCACTGGTGTTCCTC -3'	147
25	5'- GTCTGATGTGAAAGCCCACG -3'	5'- TCTCTACGCATTTCACCGCT -3'	110
26	5'- AGGGCTCAACCCTGGAATTG -3'	5'- CACTCGGAGTTCCACTCACC -3'	72
27	5'- TTAGTCAGAAGGCTGAAAGCCC -3'	5'- TACACTCGGAGTTCCACACA -3'	98
28	5'- CGCACGTAGGCGGATACTTA -3'	5'- TCCGGACTCGAGATACCCAG -3'	87
29[b]	5’- CTCAACCTCGGAACTGCCTT-3’	5’- GCCACCGGTGTTCTTCCTAA-3’	109
30	5'- TGCCATTGATACTGACGGGC -3'	5'- CTCAGCGTCAGTTACAGCCT -3'	129
31	5'- TGCCATTGATACTGACGGCT -3'	5'- CTCAGCGTCAGTTACAGCCT -3'	128
32	5'- GGTCCGTAGGCGGTCAGATA -3'	5'- GTATCAATGGCCGTTCCACC -3'	68
33	5'- GGTCCGTAGGCGGTTTAGTA -3'	5'- GTATCAATGGCCGTTCCACC -3'	68
34	5'- GGTCGCTCAACGATCAAACG -3'	5'- CGTCCATCAGCGTCAATCCA -3'	153
35	5'- CGGAAACTGCCATTGATACTATAGA -3'	5'- TGCCTCAGTGTCAATCGATCC -3'	139
36	5’- AGGTTTGCGGAATGGGTCAT -3’	5’- TTTCGAGCCTCAGCGTCAAT -3’	104
37	5’- AAGTGGGCGGAATGTGTCAT -3’	5’- TAGTGAGCTGCCTACGCAAT -3’	77
38	5’- AGTCAGAGGTGAAAGCCGGT -3’	5’- GTAAGCTGCCTTCGCAATCG -3’	144
39	5’- GGTGCGTAGGCGGCTTATTA -3’	5’- CAGTATCAAGGGCACTGCGA -3’	70

[a] Primers first published in Lozano et al (2019)

[b] Primers first published in Madeira et al (2019)

Table S4 Percent efficiency, R^2, and linear range parameters of the qPCR assays.

Assay	Percent Efficiency	R^2	Linear Range (Log_{10} copies per µL)
1	107.3	1.00	2.78-8.78
2	104.4	0.99	2.71-8.71
3	84.2	0.99	2.35-6.35
4	96.98	0.98	2.80-8.80
5	99.13	0.99	3.63-8.63
6	107.42	0.99	2.60-8.60
7	96.3	1.00	2.50-7.50
8	102.99	0.99	1.57-8.57
9	97.35	0.99	2.34-8.34
10	102.49	0.99	2.07-8.07
11	88.95	0.99	2.12-7.12
12	97.45	0.99	1.52-7.52
13	101.57	0.99	1.93-7.93
14	96.05	0.99	2.35-7.35
15	96.91	0.99	2.31-8.31
16	80.25	0.98	1.91-7.91
17	98.91	0.99	2.35-8.35
18	72.55	0.99	2.23-7.23
19	72.81	0.99	2.35-7.35
20	99.56	0.99	2.31-7.31
21	90.01	0.99	1.53-8.53
22	79.93	0.99	2.69-8.69
23	90.35	0.99	2.38-8.38
24	88.72	0.99	1.73-7.73
25	81.33	0.99	1.53-8.53
26	88.86	0.99	1.93-7.93
27	89.41	0.99	1.99-6.99
28	70.69	0.98	1.67-6.67
29	104.60	0.99	2.04-9.04
30	93.50	0.98	2.09-7.09
31	79.75	0.97	1.45-6.45
32	44.16	0.98	1.97-5.97
33	82.48	0.97	2.21-7.21
34	85.07	0.98	1.76-5.76
35	84.67	0.99	1.99-7.99
36	81.64	0.97	2.41-7.41
37	84.77	0.97	1.43-7.43
38	109.56	0.96	1.80-7.80
39	93.3	0.99	1.84-8.84

Table S5 The 16S rRNA genes mL^{-1} ($\log_{10}$ transformed shown) determined for each phylogenetic group by qPCR analysis for the microcosms amended with toluene and Cd.

Assay	Toluene only	Toluene 0.44 µM Cd	Toluene 4.4 µM Cd	Toluene 44 µM Cd	Toluene 440 µM Cd
1	6.56 ± 0.05	6.17 ± 0.35	**7.42 ± 0.15****	**7.52 ± 0.09****	**7.65 ± 0.09****
2	6.06 ± 0.30	5.37 ± 0.31*	5.74 ± 0.19	5.78 ± 0.23	5.43 ± 0.20*
3	<4.35	<4.35	<4.35	<4.35	<4.35
4	<4.80	6.09 ± 0.17**	6.04 ± 0.09**	6.28 ± 0.12**	6.98 ± 0.46**
5	<5.60	5.61 ± 0.01	<5.60	<5.60	<5.60
6	<4.60	<4.60	<4.60	<4.60	<4.60
7	6.17 ± 0.20	6.84 ± 0.26*	6.98 ± 0.69	6.89 ± 0.17**	6.73 ± 0.31
8	6.82 ± 0.13	5.46 ± 0.07**	5.60 ± 0.39**	5.80 ± 0.16**	4.32 ± 0.66**
9	6.21 ± 0.05	5.51 ± 0.40	5.20 ± 0.76	4.77 ± 0.74*	5.19 ± 0.74
10	6.21 ± 0.13	4.90 ± 0.13**	5.32 ± 0.31**	4.84 ± 0.74**	4.29 ± 0.34**
11	4.97 ± 0.17	4.59 ± 0.66	5.11 ± 0.45	<4.21**	<4.21**
12	6.00 ± 0.27	4.06 ± 0.47**	4.48 ± 0.89*	5.03 ± 0.19**	<3.52**
13	5.72 ± 0.13	5.96 ± 0.18	6.27 ± 0.50	5.74 ± 0.35	5.00 ± 0.16**
14	5.39 ± 0.09	4.88 ± 0.07**	5.15 ± 0.30	4.38 ± 0.05**	<4.35**
15	6.10 ± 0.08	6.25 ± 0.26	6.55 ± 0.72	5.94 ± 0.46	5.24 ± 0.19**
16	5.87 ± 0.19	5.32 ± 0.13*	5.60 ± 0.23	5.17 ± 0.28**	4.31 ± 0.13**
17	6.14 ± 0.07	5.30 ± 0.12**	5.58 ± 0.31*	5.71 ± 0.15*	6.10 ± 0.39
18	5.54 ± 0.07	6.11 ± 0.25*	6.38 ± 0.25*	6.64 ± 0.18**	6.90 ± 0.19**
19	5.40 ± 0.43	4.90 ± 0.24	5.04 ± 0.15	5.27 ± 0.11	5.02 ± 0.58
20	4.32 ± 0.02	<4.31	<4.31	4.38 ± 0.12	4.63 ± 0.12
21	<4.53	<4.53	<4.53	<4.53	<4.53
22	5.21 ± 0.61	5.19 ± 0.87	<4.69	5.46 ± 0.67	<4.69
23	6.41 ± 0.33	5.36 ± 0.86	5.58 ± 0.47	6.34 ± 0.55	4.94 ± 0.49*
24	5.15 ± 0.37	4.86 ± 0.23	4.84 ± 0.20	<4.73	<4.73
25	6.96 ± 0.08	6.35 ± 0.22*	6.16 ± 0.21**	6.57 ± 0.13*	6.20 ± 0.11*
26	4.91 ± 0.21	4.53 ± 0.28	4.46 ± 0.39	4.63 ± 0.21	4.97 ± 0.22
27	4.14 ± 0.09	4.05 ± 0.11	4.43 ± 0.76	4.01 ± 0.02	4.29 ± 0.27
28	4.64 ± 0.21	4.26 ± 0.30	4.72 ± 0.09	4.50 ± 0.14	4.76 ± 0.23
29	5.79 ± 0.18	5.28 ± 0.05**	5.51 ± 0.25	5.44 ± 0.29	5.44 ± 0.12
30	5.43 ± 0.46	5.35 ± 0.12	5.23 ± 0.07	3.72 ± 0.18**	4.03 ± 0.25**
31	5.49 ± 0.13	5.33 ± 0.10	5.05 ± 0.14	<3.45**	<3.45**
32	5.24 ± 0.24	5.46 ± 0.84	5.52 ± 0.85	4.63 ± 0.27*	4.58 ± 0.08*
33	5.08 ± 0.03	5.04 ± 0.77	5.48 ± 0.58	4.91 ± 0.37	5.22 ± 0.42
34	4.27 ± 0.59	4.15 ± 0.46	5.57 ± 1.12	4.06 ± 0.25	4.88 ± 0.32
35	4.77 ± 0.85	4.20 ± 0.37	5.10 ± 0.95	<3.99	<3.99
36	6.58 ± 0.13	5.85 ± 0.48	6.09 ± 0.09**	6.39 ± 0.11	4.92 ± 0.22**
37	5.62 ± 0.08	5.40 ± 0.19	5.36 ± 0.17	5.51 ± 0.03	5.56 ± 0.12
38	5.80 ± 0.04	5.12 ± 0.38*	5.37 ± 0.20*	5.24 ± 0.14**	4.46 ± 0.23**
39	6.38 ± 0.09	6.19 ± 0.35	6.38 ± 0.31	6.06 ± 0.04**	4.78 ± 0.37**

* Demonstrates significant difference (*P*<0.05) between value and the toluene only control (student t-test)
** Demonstrates significant difference (*P*<0.01) between value and the toluene only control (student t-test)
Values are average of the triplicate microcosms ± standard deviations.
Red text designates statistically lower numbers compared to the no-metals control; blue text designates statistically higher numbers compared to the no-metals control. Values over 7 log units 16S rRNA genes mL^{-1} are bolded.

Table S6 The 16S rRNA genes mL^{-1} (log$_{10}$ transformed shown) determined for each phylogenetic group by qPCR analysis for the microcosms amended with toluene and Pb.

Assay	Toluene only	Toluene 0.24 µM Pb	Toluene 2.4 µM Pb	Toluene 24 µM Pb	Toluene 240 µM Pb
1	6.56 ± 0.05	5.99 ± 0.23*	6.23 ± 0.46	6.16 ± 0.26	6.14 ± 0.71
2	6.06 ± 0.30	5.45 ± 0.15*	6.47 ± 0.66	6.52 ± 0.27	6.85 ± 0.33*
3	<4.35	<4.35	<4.35	<4.35	5.94 ± 0.09 **
4	<4.80	<4.80	<4.80	<4.80	<4.80
5	<5.60	<5.60	6.40 ± 1.33	6.24 ± 0.84	6.31 ± 0.50
6	<4.60	<4.60	<4.60	5.19 ± 1.02	5.32 ± 0.55
7	6.17 ± 0.20	6.50 ± 0.30	**7.35 ± 0.60***	**7.44 ± 0.17****	**7.45 ± 0.17****
8	6.82 ± 0.13	5.92 ± 0.46*	6.68 ± 0.59	**7.23 ± 0.19***	**7.28 ± 0.20***
9	6.21 ± 0.05	5.50 ± 0.41*	6.26 ± 0.48	6.59 ± 0.07**	6.77 ± 0.15**
10	6.21 ± 0.13	5.36 ± 0.47*	5.97 ± 0.59	6.42 ± 0.16	6.43 ± 0.18
11	4.97 ± 0.17	4.41 ± 0.35	5.01 ± 0.45	4.85 ± 0.68	5.09 ± 0.87
12	6.00 ± 0.27	4.27 ± 0.60*	5.22 ± 1.12	5.58 ± 0.72	5.32 ± 0.86
13	5.72 ± 0.13	5.67 ± 0.39	6.04 ± 0.36	6.23 ± 0.05**	6.10 ± 0.18
14	5.39 ± 0.09	4.53 ± 0.31*	5.03 ± 0.59	4.75 ± 0.59	4.71 ± 0.62
15	6.10 ± 0.08	5.59 ± 0.37	6.17 ± 0.10	6.21 ± 0.04	6.01 ± 0.02
16	5.87 ± 0.19	4.91 ± 0.13**	5.26 ± 0.25*	5.07 ± 0.81	4.91 ± 0.74
17	6.14 ± 0.07	5.63 ± 0.20*	6.51 ± 0.51	6.59 ± 0.18*	6.60 ± 0.41
18	5.54 ± 0.07	6.19 ± 0.30*	**7.13 ± 0.73***	**7.16 ± 0.15****	**7.28 ± 0.12****
19	5.40 ± 0.43	5.05 ± 0.39	5.50 ± 0.24	5.64 ± 0.29	5.21 ± 0.21
20	4.32 ± 0.02	<4.31	4.38 ± 0.10	4.53 ± 0.09	<4.31
21	<4.53	4.69 ± 0.28	<4.53	<4.53	<4.53
22	5.21 ± 0.61	5.24 ± 0.54	<4.69	5.48 ± 1.37	6.61 ± 0.19*
23	6.41 ± 0.33	5.19 ± 0.54	6.36 ± 0.21	6.49 ± 0.38	6.36 ± 0.24
24	5.15 ± 0.37	<4.73	5.03 ± 0.28	5.22 ± 0.53	5.36 ± 0.40
25	6.96 ± 0.08	6.11 ± 0.27**	**7.13 ± 0.39**	**7.17 ± 0.29**	**7.13 ± 0.12**
26	4.91 ± 0.21	4.23 ± 0.28	4.73 ± 0.31	4.81 ± 0.12	4.62 ± 0.24
27	4.14 ± 0.09	<3.99*	4.44 ± 0.14*	4.34 ± 0.36	4.02 ± 0.03
28	4.64 ± 0.21	4.55 ± 0.33	4.75 ± 0.29	4.94 ± 0.07	4.58 ± 0.13
29	5.79 ± 0.18	4.97 ± 0.27*	5.79 ± 0.24	5.84 ± 0.06	5.70 ± 0.08
30	5.43 ± 0.46	5.08 ± 0.01	5.55 ± 0.39	4.73 ± 1.04	5.56 ± 0.40
31	5.49 ± 0.13	4.98 ± 0.17	5.50 ± 0.26	5.14 ± 1.06	5.44 ± 0.85
32	5.24 ± 0.24	5.13 ± 0.85	5.21 ± 0.70	5.67 ± 0.69	5.90 ± 0.06*
33	5.08 ± 0.03	4.98 ± 0.69	5.24 ± 0.16	5.16 ± 0.17	4.97 ± 0.06*
34	4.27 ± 0.59	4.47 ± 1.13	4.93 ± 1.02	4.12 ± 0.29	3.84 ± 0.09
35	4.77 ± 0.85	4.53 ± 0.51	4.84 ± 0.77	4.23 ± 0.28	4.41 ± 0.72
36	6.58 ± 0.13	5.87 ± 0.31*	6.49 ± 0.50	6.43 ± 0.29	6.03 ± 0.50
37	5.62 ± 0.08	5.30 ± 0.40	5.68 ± 0.36	6.00 ± 0.23	5.63 ± 0.39
38	5.80 ± 0.04	4.83 ± 0.21**	5.49 ± 0.19*	5.35 ± 0.18*	5.42 ± 0.10**
39	6.38 ± 0.09	5.78 ± 0.26*	6.23 ± 0.40	6.18 ± 0.13	6.20 ± 0.21

* Demonstrates significant difference (P<0.05) between value and the toluene only control (student t-test)
** Demonstrates significant difference (P<0.01) between value and the toluene only control (student t-test)
Values are average of the triplicate microcosms ± standard deviations.
Red text designates statistically lower numbers compared to the no-metals control; blue text designates statistically higher numbers compared to the no-metals control. Values over 7 log units 16S rRNA genes mL^{-1} are bolded.

Table S7 The 16S rRNA genes mL^{-1} (log_{10} transformed shown) determined for each phylogenetic group by qPCR analysis for the microcosms amended with benzene and Cd.

Assay	Benzene only	Benzene 0.44 µM Cd	Benzene 4.4 µM Cd	Benzene 44 µM Cd	Benzene 440 µM Cd
1	6.57 ± 0.33	6.58 ± 0.82	**7.18 ± 0.31**	**7.52 ± 0.34***	**7.87 ± 0.23****
2	6.79 ± 0.37	6.02 ± 0.16*	6.42 ± 0.60	6.42 ± 0.35	6.02 ± 0.22*
3	<4.35	<4.35	<4.35	<4.35	<4.35
4	<4.80	<4.80	<4.80	<4.80	<4.80
5	<5.60	5.76 ± 0.18	5.82 ± 0.29	5.98 ± 0.38	5.70 ± 0.09
6	<4.60	<4.60	<4.60	5.48 ± 1.04	4.64 ± 0.07
7	6.84 ± 0.44	6.29 ± 0.67	**7.10 ± 0.60**	**7.79 ± 0.45**	6.0 ± 0.13*
8	**7.00 ± 0.12**	6.84 ± 0.72	**7.50 ± 0.23***	**7.41 ± 0.13****	5.67 ± 0.15**
9	6.33 ± 0.19	6.26 ± 0.50	6.70 ± 0.17	6.79 ± 0.15	6.25 ± 0.20
10	6.56 ± 0.09	6.36 ± 0.65	6.89 ± 0.21	6.82 ± 0.09*	5.73 ± 0.18**
11	6.55 ± 0.39	5.22 ± 0.44*	6.00 ± 0.57	6.38 ± 0.29	<4.21**
12	5.09 ± 0.78	5.04 ± 0.07	5.08 ± 0.72	4.95 ± 1.13	3.70 ± 0.16*
13	**7.24 ± 0.53**	6.52 ± 0.63	**7.31 ± 0.91**	**7.48 ± 0.69**	5.88 ± 0.40*
14	6.30 ± 0.61	5.81 ± 0.55	6.73 ± 0.62	6.67 ± 0.71	5.04 ± 0.49*
15	**7.07 ± 0.14**	6.60 ± 0.38	**7.27 ± 0.19**	**7.24 ± 0.13**	6.28 ± 0.56
16	6.40 ± 0.51	6.14 ± 0.39	**7.00 ± 0.42**	6.80 ± 0.66	5.52 ± 0.62
17	6.44 ± 0.13	6.62 ± 0.39	6.65 ± 0.15	6.59 ± 0.12	6.99 ± 0.36
18	5.75 ± 0.06	5.89 ± 0.11	5.89 ± 0.16	5.74 ± 0.21	5.83 ± 0.29
19	5.17 ± 0.14	5.72 ± 0.18*	5.55 ± 0.18*	6.04 ± 0.15**	5.71 ± 0.33
20	4.60 ± 0.21	4.33 ± 0.04	4.58 ± 0.32	4.58 ± 0.15	5.40 ± 0.14**
21	6.28 ± 0.16	4.95 ± 0.73	5.04 ± 0.88	<4.53**	<4.53**
22	6.28 ± 0.13	5.39 ± 0.61	5.07 ± 0.63	4.77 ± 0.13**	<4.69**
23	6.23 ± 0.61	5.91 ± 0.01	5.54 ± 0.62	5.83 ± 0.28	6.64 ± 0.24
24	5.08 ± 0.50	4.89 ± 0.15	4.76 ± 0.38	<4.73	4.87 ± 0.24
25	6.36 ± 0.13	**7.03 ± 0.08****	6.62 ± 0.08*	6.30 ± 0.20	6.42 ± 0.13
26	4.96 ± 0.31	4.86 ± 0.15	4.85 ± 0.31	5.04 ± 0.14	5.33 ± 0.09
27	4.99 ± 0.29	4.42 ± 0.75	5.95 ± 0.29*	4.79 ± 0.68	4.92 ± 0.03
28	4.78 ± 0.12	4.92 ± 0.22	4.94 ± 0.09	4.85 ± 0.09	5.02 ± 0.18
29	6.11 ± 0.26	5.79 ± 0.11	5.84 ± 0.11	5.67 ± 0.19	5.59 ± 0.07*
30	4.02 ± 0.23	4.77 ± 0.88	4.17 ± 0.61	4.09 ± 0.06	3.91 ± 0.15
31	<3.45	3.87 ± 0.37	3.78 ± 0.31	<3.45	<3.45
32	5.15 ± 0.10	5.19 ± 0.30	5.43 ± 0.30	5.73 ± 0.77	4.98 ± 0.11
33	5.65 ± 0.26	5.61 ± 0.33	5.67 ± 0.33	5.78 ± 0.14	5.37 ± 0.20
34	5.00 ± 1.25	3.77 ± 0.02	4.16 ± 0.44	4.20 ± 0.34	4.38 ± 0.54
35	6.28 ± 0.21	5.05 ± 1.14	6.33 ± 0.21	5.85 ± 0.25	<3.99**
36	6.96 ± 0.31	6.86 ± 0.13	6.93 ± 0.15	6.96 ± 0.03	5.37 ± 0.22**
37	6.09 ± 0.18	5.40 ± 0.05**	5.56 ± 0.04**	5.90 ± 0.08	5.13 ± 0.23**
38	5.62 ± 0.11	5.63 ± 0.15	5.39 ± 0.62	5.63 ± 0.13	5.15 ± 0.11**
39	6.12 ± 0.37	5.99 ± 0.14	6.17 ± 0.09	5.78 ± 0.37	4.63 ± 0.41**

* Demonstrates significant difference ($P<0.05$) between value and the toluene only control (student t-test)
** Demonstrates significant difference ($P<0.01$) between value and the toluene only control (student t-test)
Values are average of the triplicate microcosms ± standard deviations.
Red text designates statistically lower numbers compared to the no-metals control; blue text designates statistically higher numbers compared to the no-metals control. Values over 7 log units 16S rRNA genes mL^{-1} are bolded.

Table S8 The 16S rRNA genes mL^{-1} (log_{10} transformed shown) determined for each phylogenetic group by qPCR analysis for the microcosms amended with benzene and Pb.

Assay	Benzene only	Benzene 0.24 µM Pb	Benzene 2.4 µM Pb	Benzene 24 µM Pb	Benzene 240 µM Pb
1	6.57 ± 0.33	**7.20 ± 0.08***	**7.20 ± 0.21***	**7.48 ± 0.33***	**7.85 ± 0.32****
2	6.79 ± 0.37	6.62 ± 0.41	**7.06 ± 0.09**	**7.03 ± 0.18**	6.97 ± 0.27
3	<4.35	<4.35	<4.35	<4.35	<4.35
4	<4.80	4.80 ± 0.01	<4.80	4.90 ± 0.18	<4.80
5	<5.60	5.73 ± 0.08*	5.75 ± 0.13	6.12 ± 0.39	<5.60
6	<4.60	4.68 ±0.14	<4.60	5.05 ± 0.43	<4.60
7	6.84 ± 0.44	6.91 ± 0.20	**7.23 ± 0.30**	**7.61 ± 0.04***	**7.71 ± 0.16***
8	**7.00 ± 0.12**	**7.13 ± 0.29**	**7.39 ± 0.46**	6.89 ± 0.15	**7.10 ± 0.33**
9	6.33 ± 0.19	6.38 ± 0.29	6.53 ± 0.40	6.08 ± 0.09	6.64 ± 0.02
10	6.56 ± 0.09	6.54 ± 0.29	6.98 ± 0.47	6.43 ± 0.11	6.58 ± 0.27
11	6.55 ± 0.39	6.17 ± 0.56	6.10 ± 0.11	4.50 ± 0.47*	5.74 ± 0.16*
12	5.09 ± 0.78	4.75 ± 1.2	5.61 ± 0.01	4.74 ± 0.68	5.43 ± 0.99
13	**7.24 ± 0.53**	6.48 ± 0.05	**7.32 ± 0.82**	5.27 ± 1.23	5.52 ± 0.51*
14	6.30 ± 0.61	5.42 ± 0.46	6.46 ± 0.77	5.20 ± 0.67	5.66 ± 0.48
15	**7.07 ± 0.14**	6.89 ± 0.44	**7.23 ± 0.06**	6.85 ± 0.54	**7.02 ± 0.10**
16	6.40 ± 0.51	5.74 ± 0.37	6.61 ± 0.85	5.50 ± 0.93	6.11 ± 0.16
17	6.44 ± 0.13	6.64 ± 0.11	6.72 ± 0.18	6.60 ± 0.18	6.64 ± 0.16
18	5.75 ± 0.06	5.71 ± 0.06	5.88 ± 0.10	5.79 ± 0.12	5.69 ± 0.19
19	5.17 ± 0.14	5.66 ± 0.29	5.86 ± 0.24*	5.75 ± 0.29*	5.37 ± 0.46
20	4.60 ± 0.21	4.46 ± 0.27	4.58 ± 0.24	<4.31	4.57 ± 0.33
21	6.28 ± 0.16	<4.53**	<4.53**	<4.53**	<4.53**
22	6.28 ± 0.13	<4.69**	5.09 ± 0.69	5.54 ± 0.77	5.51 ± 0.74
23	6.23 ± 0.61	6.51 ± 0.22	6.52 ± 0.52	6.47 ± 0.24	5.62 ± 0.19
24	5.08 ± 0.50	5.63 ± 0.22	5.55 ± 0.69	5.38 ± 0.59	4.76 ± 0.06
25	6.36 ± 0.13	6.61 ± 0.31	6.54 ± 0.24	**7.00 ± 0.23***	6.59 ± 0.18
26	4.96 ± 0.31	4.94 ± 0.10	5.14 ± 0.05	4.92 ± 0.11	4.81 ± 0.10
27	4.99 ± 0.29	5.15 ± 0.78	5.68 ± 0.47	4.10 ± 0.19*	5.21 ± 0.74
28	4.78 ± 0.12	4.95 ± 0.16	4.88 ± 0.17	4.82 ± 0.14	4.75 ± 0.11
29	6.11 ± 0.26	5.88 ± 0.08	5.90 ± 0.12	5.90 ± 0.04	5.77 ± 0.16
30	4.02 ± 0.23	3.96 ± 0.28	4.10 ± 0.12	4.18 ± 1.19	4.53 ± 0.68
31	<3.45	<3.45	<3.45	3.80 ± 0.46	4.14 ± 0.90
32	5.15 ± 0.10	5.35 ± 0.28	5.25 ± 0.03	5.19 ± 0.12	5.34 ± 0.48
33	5.65 ± 0.26	5.96 ± 0.22	6.46 ± 0.58	5.33 ± 0.10	5.41 ± 0.25
34	5.00 ± 1.25	4.66 ± 1.33	4.16 ± 0.43	3.82 ± 0.11	3.96 ± 0.19
35	6.28 ± 0.21	6.30 ± 0.14	6.29 ± 0.22	4.34 ± 0.60**	6.06 ± 0.25
36	6.96 ± 0.31	6.85 ± 0.23	6.95 ± 0.22	6.86 ± 0.20	6.65 ± 0.31
37	6.09 ± 0.18	5.75 ± 0.15	6.16 ± 0.20	5.59 ± 0.28	5.73 ± 0.14
38	5.62 ± 0.11	5.73 ± 0.05	**7.05 ± 0.09****	5.69 ± 0.12	5.49 ± 0.17
39	6.12 ± 0.37	5.82 ± 0.25	5.49 ± 0.39	6.02 ± 0.38	5.79 ± 0.31

* Demonstrates significant difference (*P*<0.05) between value and the toluene only control (student t-test)
** Demonstrates significant difference (*P*<0.01) between value and the toluene only control (student t-test)
Values are average of the triplicate microcosms ± standard deviations.
Red text designates statistically lower numbers compared to the no-metals control; blue text designates statistically higher numbers compared to the no-metals control. Values over 7 log units 16S rRNA genes mL^{-1} are bolded.

References

Edgar, R. C. (2004). MUSCLE: multiple sequence alignment with high accuracy and high throughput. *Nucleic Acids Research* 32 (5): 1792-1797.

Felsenstein, J. (1985). Confidence limits on phylogenies: An approach using the bootstrap. *Evolution* 39: 783-791.

Kumar, S., Stecher G., Li, M., Knyaz, C., and K. Tamura (2018). MEGA X: Molecular Evolutionary Genetics Analysis across multiple computing platforms. *Molecular Biology and Evolution* 35:1547-1549.

Lozano, T. M., A. L. McCutchan, M. J. Krzmarzick (2019). Hydraulic fracturing fluid compositions induce different enrichment of soil bacterial communities. Environmental Engineering Science 36 (4): 385-395.

Madeira, C. L., K. V. Jog, E. T. Vanover, M. D. Brooks, D. K. Taylor, R. Sierra-Alvarez, L. A. Waidner, J. C. Spain, M. J. Krzmarzick and J. A. Field (2019). Microbial enrichment culture responsible for the complete oxidation of 3-amino-1,2,3-traizol-5-one (ATO), the reduced daughter product of the insensitive munitions compound 3-nitro-1,2,4-triazol-5-one (NTO). Environ. Sci. Technol. 53 (21): 12648-12656.

Saitou, N., and M. Nei (1987). The neighbor-joining method: A new method for reconstructing phylogenetic trees. *Molecular Biology and Evolution* 4: 406-425.

Tamura, K., Nei, M., and S. Kumar (2004). Prospects for inferring very large phylogenies by using the neighbor-joining method. *Proceedings of the National Academy of Sciences (USA)* 101: 11030-11035.
